# Can COVID-19 herd immunity be achieved at a city level?

**DOI:** 10.1371/journal.pone.0299574

**Published:** 2024-05-29

**Authors:** Yuval Arbel, Yifat Arbel, Amichai Kerner, Miryam Kerner

**Affiliations:** 1 Sir Harry Solomon School of Economics and Management, Western Galilee College, Acre, Israel; 2 Department of Mathematics, Bar Ilan University, Ramat Gan, Israel; 3 Faculty of Social Sciences, Banking and Finance Program, Bar Ilan University, Ramat Gan, Israel; 4 The Ruth and Bruce Rapoport Faculty of Medicine, Technion – Israel Institute of Technology, Haifa, Israel; 5 Department of Dermatology, Emek Medical Center, Afula, Israel; University of California Davis School of Medicine, UNITED STATES

## Abstract

We propose a new approach to estimate the vaccination rates required to achieve herd immunity against SARS-COV2 virus at a city level. Based on information obtained from the Israeli Ministry of Health, we estimate two separate quadratic models, one for each dose of the BNT162b2 mRNA Pfizer vaccine. The dependent variable is the scope of morbidity, expressed as the number of cases per 10,000 persons. The independent variables are the first and second vaccination rates and their squares. The outcomes corroborate that herd immunity is achieved in the case that 71 percent of the urban population is vaccinated, and the minimum anticipated scope of morbidity is approximately 5 active COVID-19 cases per 10,000 persons, compared to 53–67 cases per 10,000 persons for zero vaccination rate. Findings emphasize the importance of vaccinations and demonstrate that urban herd immunity may be defined as a situation in which people continue to interact, yet the COVID-19 spread is contained. This, in turn, might prevent the need for lockdowns or other limitations at the city level.

## Introduction

Accumulated evidence corroborates the effectiveness of the BNT162b2 vaccine against SARS-COV-2 virus. Based on a matched-paired sample of 596,618 vaccinated and 596,618 unvaccinated individuals with similar characteristics, Dagan et al. (2021) [1] tested the effectiveness of the vaccine in Israel after the first and second doses. Yet, the extent of vaccination required to generate herd immunity against SARS-COV2 remains an open question.

The notion of herd immunity is used to depict the threshold of immune individuals that will lead to a decrease in disease incidence (Clemente-Suárez et al., 2020 [2]). Herd immunity is a dynamic notion that may vary from one disease to other and from one region to a different one (Kalish et al (2021) [3]; Barker et al. (2021) [4]; Pei et al. (2021) [5]). This characteristic makes it difficult to estimate the threshold required to achieve herd immunity against SARS-COV2.

The objective of the current study is to propose a new approach to estimate the vaccination rates required to achieve herd immunity against SARS-COV2 virus at a city level. Based on information obtained from the Israeli Ministry of Health [6], we estimate two separate quadratic models, one for each dose of the BNT162b2 mRNA Pfizer vaccine. The dependent variable is the scope of morbidity, expressed as the number of cases per 10,000 persons. The independent variables are the first and second vaccination rates and their squares. Findings demonstrate that vaccination rate of 71 percent of the urban population is anticipated to yield the minimum scope of morbidity (approximately five COVID-19 cases per 10,000 persons).

### Stylized facts on pandemic spreads and herd immunity

Coronavirus disease (COVID-19) is an infectious disease caused by the SARS-CoV-2 virus. The most common symptoms of the disease are cough, fever, loss of taste or smell and tiredness (World Health Organization [7]). The Delay between symptom onset and access to intensive care is essential to prevent clinical worsening for different infectious diseases. Referring to the COVID19 pandemic, patients with a long delay between symptom onset and hospital admission had higher body mass index, were younger, and were more frequently admitted to intensive care unit (Dananché et al., 2022 [8]).

Herd immunity refers to a: “state in which a large proportion of a population is able to repel an infectious disease, thereby limiting the extent to which the disease can spread from person to person. Herd immunity can be conferred through natural immunity, previous exposure to the disease, or vaccination. An entire population does not need to be immune to attain herd immunity. Rather, herd immunity can occur when the population density of persons who are susceptible to infection is sufficiently low so as to minimize the likelihood of an infected individual coming in contact with a susceptible individual.” (Lee, 2016 [9]). According to Clemente-Suárez et al. (2020) [2], the concept of herd immunity is used to describe the threshold of immune individuals that will lead to a decrease in disease incidence.

Referring to different strains of the SARS-CoV2 virus, on November 23, 2021, the Institute of Infectious Diseases in South Africa identified a new variant of Corona that was named Omicron. Its scientific name is BA1. This strain spread at breakneck speed throughout the world and pushed all the strains that preceded it to the margins, including the delta strain. This is a strain that has multiple mutations, and dozens of them are related to the production of the spike protein—the protein that is responsible for the penetration of the corona virus into cells in the human body, and against which the current vaccines work. The sub-strains BA4 and BA5—both mutations of the omicron—were dominant in the spring of 2022. They were preceded by strain BA2 which was dominant in early 2022.

Like any new breed—these breeds also carried new mutations that improved their survivability. For example, the BA2 strain became dominant in Israel at the beginning of 2022 due to its improved infectivity: it was about 30% more contagious than the original Omicron (BA1), which was also an especially contagious strain compared to the first strains at the beginning of the epidemic. Even before that, many other varieties appeared, including the Indian, British and South African varieties (Clalit Healthcare services website (Hebrew) [10]).

The dominant strain during the study period was Omicron with its various mutations. The information given by the Clalit Healthcare Services (Clalit Healthcare provides services to 50% of the total Israeli population) refers only to the Omicron strain without specifically referring to each mutation separately. With regard to the other strains, given that the omicron dominated significantly, the other strains hardly appeared.

S1 and S2 Appendices in the supporting information exhibit the herd immunity required to reduce the level of infection in different diseases. At the lowest end, the required proportion of immuned persons to generate herd immunity against Andes hantavirus and influenza (seasonal strains) are only 16% and 23%, respectively. At the highest end, the thresholds against chickenpox (varicella) and measles are 90–94% of the population. Based on these two extremes, COVID-19 is closer to the upper threshold, with 75–80% threshold to the Alpha variant, 58–71% threshold to the ancestral strain, and 80% threshold to the Delta variant.

The fact that herd immunity is a dynamic concept that may vary from one disease to another and from one region to another is also reported by Kalish et al (2021) [3]; Barker et al. (2021) [4]; Pei et al. (2021) [5]. Kalish et al. (2021) [3] estimated ratios of between 1.8 and 12.2 for different regions of the U.S. as of the summer of 2020, with recent estimates closer to 4. In this regard, we propose a new approach to estimate the vaccination rates required to achieve herd immunity against SARS-COV2 virus at a city level.

### Description of data

The raw dataset is given in supporting information as S1 File. To replicate the results–one should use the Stata software package and modify the first row of the do file that begins with the “cd” command (S2 File in the supporting information) to the directory where the raw data file is included. The output file is given as a log file converted to pdf. in S3 File.

#### Descriptive statistics

Tables 1 and 2 exhibit the descriptive statistics of the variables incorporated subsequently in the regression analysis. The table refers to 132 Israeli cities and towns, which comprise 5,964,295 residents (above $\frac{5,964,295}{9,217,000}=64.71\%$ of the entire Israeli population), where the number of active COVID-19 cases per 10,000 persons ≥0.

**Table 1:** Description of variables.

Variable	Description
Cases_per_10,000	COVID-19 active cases divided by the population of the city and multiplied by 10,000
Second vaccination	Percent of persons that took the second dose of the COVID-19 Pfizer vaccine multiplied by 100
First_vaccination	Percent of persons that took the first dose of the COVID-19 Pfizer vaccine multiplied by 100
Diff second first	The difference between the second and first vaccination

**Table 2:** Descriptive statistics.

Variable	Obs	Mean	Median	Std. Dev.	Min	Max	99% confidence interval
Cases_per_10,000	132	9.656	2.350	19.150	0	135.4	[5.299, 14.013]
Second_vaccination	132	56.867	60.905	16.983	6.83	90	[53.000, 60.731]
First_vaccination	132	63.269	67.635	16.841	8.32	90	[60.369, 66.169]
Diff second first	132	-6.402	-5.745	2.544	-15.74	0	[-6.840, -5.963]

Notes: The table refers to 132 Israeli cities and towns, which cover above 5,964,295 inhabitants (above $\frac{5,964,295}{9,217,000}=64.71\%$ of the entire Israeli population). 99% confidence intervals are given in square brackets.

**Fig 1:**
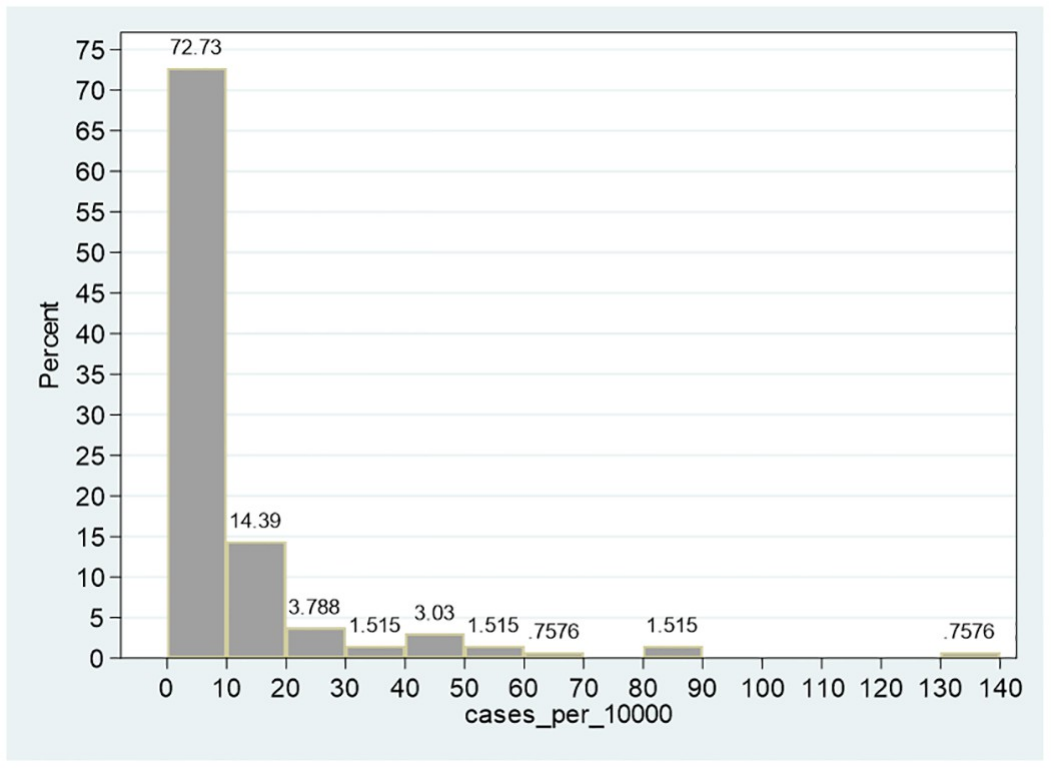
Distribution of COVID-19 cases per 10,000 persons. Notes: The figure describes the continuous distribution of active COVID-19 cases per 10,000 persons. The vertical axis (percent) is the relative prevalence (the area of each rectangular with width of 10 COVID-19 cases per 10,000 persons). The skewness of the distribution is 3.61 and the null hypothesis of symmetrical distribution is clearly rejected (adjusted calculated chi-square with two degrees of freedom of 91.98 compared to 1% critical value of 9.21).

Referring to COVID-19 active cases normalized by population size (cases_per_10000), the sample mean is 9.66 and the sample median is 2.35 COVID-19 cases per 10,000 persons. The implication is a right-tailed distribution, namely, few cities with high COVID-19 infection rates and many cities with low COVID-19 infection rates. This pattern is demonstrated in Fig 1, which gives the histogram of the COVID-19 cases per 10,000 persons. The scope of morbidity in 72.73 percent of the entire sample of 132 cities is 0–10 cases per 10,000 persons, in 14.39 percent– 10–20 cases per 10,000 persons, and in 12.88 percent (the complementary to 100 percent)– 20–140 cases per 10,000 persons. The skewness of the distribution is positive (+3.61) and the null hypothesis of symmetrical distribution is clearly rejected (adjusted calculated chi-square with two degrees of freedom of 91.98 compared to 1% critical value of 9.21). By comparison, referring to the United States, Beare and Toda (2020) [11] show similar distributions in COVID-19 growth rates (page 5 in Beare and Toda, 2020 [11]).

Other important features of cases_per_10000 are the standard deviation (19.150), the minimum (zero active cases per 10000 persons), and the maximum (135.4 active cases per 10,000 persons). The 99% confidence interval in Table 1 [5.299, 14.013] demonstrates that the null hypothesis of zero COVID-19 active cases per 10,000 persons is clearly rejected.

**Fig 2:**
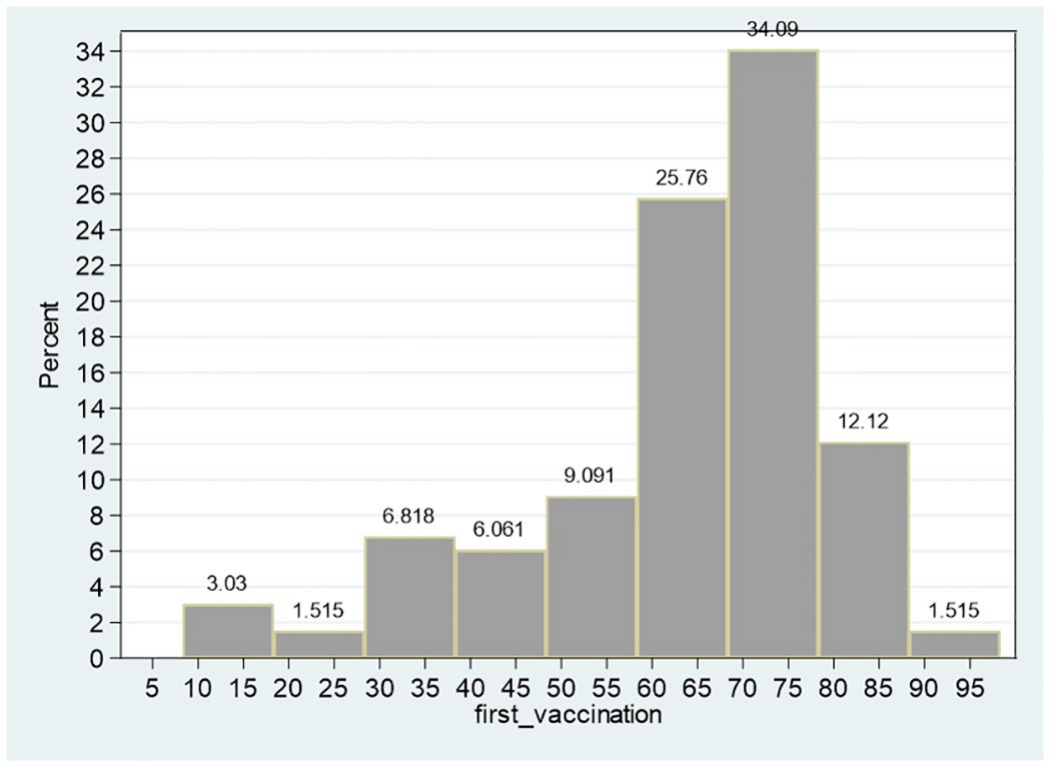
Distribution of percent of vaccinated persons first vaccination. Notes: The skewness of the distribution is −1.25 and the null hypothesis of symmetrical distribution is clearly rejected (adjusted calculated chi-square with two degrees of freedom of 23.70 compared to 1% critical value of 9.21).

**Fig 3:**
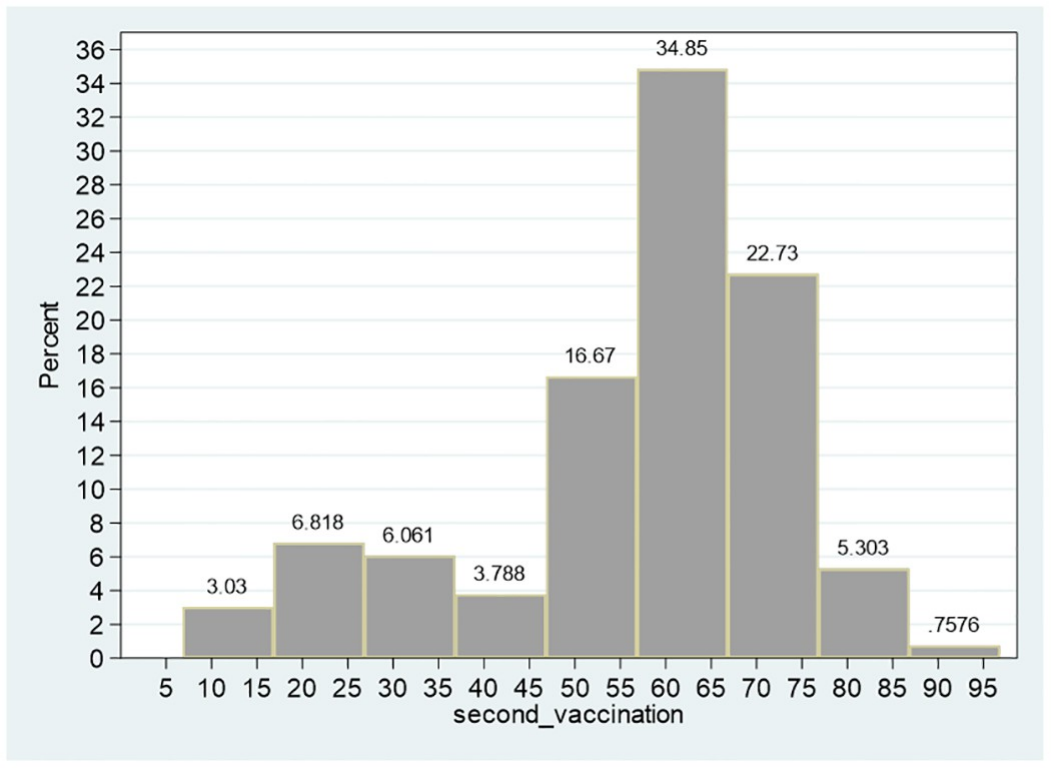
Distribution of percent of vaccinated persons second vaccination. Notes: The skewness of the distribution is −0.98 and the null hypothesis of symmetrical distribution is clearly rejected (adjusted calculated chi-square with two degrees of freedom of 15.75 compared to 1% critical value of 9.21).

Referring to the variables second_vaccination and first_vaccination (percent of persons who received the second and first dose of the BNT162b2 mRNA Covid-19 Pfizer vaccine), the sample means are 56.87, 63.269 and the sample medians are 60.905, 67.635, respectively. Given that for both variables, the median is greater than the mean, both distributions are expected to be left-tailed (Figs 1–3). The skewness of both distributions are negative (−1.25, −0.98, respectively) and the separate null hypotheses of symmetrical distributions are clearly rejected (adjusted calculated chi-square with two degrees of freedom of 23.70, 15.75 compared to 1% critical value of 9.21).

The mean, median and the 99% confidence intervals of these two variables ([53.000, 60.731] for the second vaccination and [60.369, 66.169] for the first vaccination), indicate a reduction in the percent of population who took the second dose following a shift from the first to the second vaccination, given approximately two-three weeks later. For instance, based on the 99% confidence intervals, while the null hypothesis of 65 prevalence of the city population, who took the second dose is clearly rejected, the same hypothesis is not rejected for the first dose. Referring to the power of the test for the first dose, even if the confidence interval is reduced to 90% ([60.841, 65.697]), the null hypothesis of equality of the mean to 65 percent is not rejected This outcome is further corroborated by testing the null hypothesis of equality of means for matched pair. The average difference is −6.402, and the 99% confidence interval is [−6.840, −5.963]–indicating that the null hypothesis of zero difference is clearly rejected. Even one-sided null hypothesis that the difference is greater from or equal to zero is clearly rejected at the 10% significance level (calculated *t* value with 131 degrees of freedom of −28.911).

The implication is that on average, fewer people took both vaccinations compared to those who took only one vaccination. Still, as demonstrated in the subsequent section, there is high collinearity between these two variables (second_vaccination and first_vaccination). With the exception of about 6%, who received only one dose of the vaccine, the remainder of the vaccinated population (94%) received two doses of the vaccine.

#### Collinearity diagnostics and the Pearson correlation matrix

Column (1) of Table 3A reports the regression outcomes obtained from the following empirical model:

$$Cases\_per\_10,000=\alpha_{0}^{\prime }+\alpha_{1}^{\prime }first\_vaccination+\alpha_{2}^{\prime }second\_vaccination+\mu_{1}^{\prime }$$ (1)

where *Cases*_*per*_10,000 is the dependent variable, *first*_*vaccination, second*_*vaccination* are the independent variables, $\alpha_{0}^{\prime },\alpha_{1}^{\prime },\alpha_{2}^{\prime }$ are parameters and $\mu_{1}^{\prime }$ is the stochastic random disturbance term.

**Table 3:** Collinearity and Pearson correlation matrix.

A. Regression Analysis (132 Cities and Towns)			
VARIABLES	(1)	(2)	(3)
	cases_per_10,000	cases_per_10,000	cases_per_10,000
Constant	39.82***	34.12***	37.87***
	(1.26×10^−7^)	(4.47×10^−9^)	(4.14×10^−9^)
Second_vaccination	0.314	-0.430***	–
	(0.608)	(6.41×10^−6^)	–
First_vaccination	-0.759	–	-0.446***
	(0.219)	–	(3.33×10^−6^)
Observations	132	132	132
R-squared	0.155	0.145	0.154
Calculated F-Value	11.87***	22.13***	23.62***
d.f numerator	2	1	1
d.f Denominator	129	130	130
1% Critical F-Value	4.774	6.834	6.834
B. Pearson Correlation Matrix (132 Cities and Towns)			
	cases_per_10,000	Second_vaccination	First_vaccination
cases_per_10,000	1.0000		
	132		
Second vaccination	-0.3814***	1.0000	
	(<0.01)		
First_vaccination	-0.3921***	0.9887***	1.0000
	(<0.01)	(<0.01)	

Notes: P-values are given in parentheses. At the bottom of table, the investigated null hypothesis is zero correlation and the alternative hypothesis is correlation different from zero.

*p<0.1;

**p<0.05;

***p<0.01.

Interestingly, referring to each of the two independent variables, the separate null hypotheses of equality of their coefficients to zero is not rejected empirically (*p* = 0.608 for ${\hat{\alpha }}_{1}^{\prime }$ and *p* = 0.219 for ${\hat{\alpha }}_{2}^{\prime }$, where the circumflexes denote the estimated parameters). Moreover, the sign of ${\hat{\alpha }}_{1}^{\prime }$ is positive, indicating a rise in anticipated extent of COVID-19 morbidity following an increase in vaccination rates.

Based solely on these two statistical tests, one could argue that a rise in the vaccination rate does not influence the anticipated scope of COVID-19 morbidity. Yet, note the rejection of the joint null hypothesis that both coefficients are equal to zero (Calculated *F*(2,129) = 11.87 compared to 1% critical *F*(2,129) = 4.773).

This alleged contradiction between the outcomes, obtained from the *F*-test of the regression significance and the *t*-test of each coefficient separately, is a classical indicator of high collinearity between the two explanatory variables (e.g., Johnston and Dinardo, 1997 [12]: 88–89; Ramanathan, 2002 [13]: 214–220). Indeed, as Table 3B indicates, the Pearson correlation between second- and first vaccination is 0.9887 and the null hypothesis of equality of this Pearson correlation to zero is clearly rejected.

Columns (2) and (3) in Table 3A demonstrate that if we run separate regressions based on the following empirical models: $Cases\_per\_10,000=\alpha_{0}^{\prime \prime }+\alpha_{1}^{\prime \prime }first\_vaccination+\mu_{1}^{\prime \prime }$ and $Cases\_per\_10,000=\alpha_{0}^{\prime \prime \prime }+\alpha_{1}^{\prime \prime \prime }second\_vaccination+\mu_{1}^{\prime \prime \prime }$, and as anticipated, each of the two explanatory variables are negatively correlated with the prevalence of COVID-19 morbidity. The estimated coefficients are: ${\hat{\alpha }}_{1}^{\prime \prime }=-0.430$ (*p* = 6.41 × 10^−6^) and ${\hat{\alpha }}_{1}^{\prime \prime \prime }=-0.446$ (*p* = 3.33 × 10^−6^). A $2.326=\frac{1}{0.430}$ percent *rise* in the prevalence of second vaccination and a $2.242=\frac{1}{0.446}$ percent rise in the prevalence of the first vaccination is associated with an anticipated *fall* by one COVID-19 active case per 10,000 persons. This conclusion is further verified at the bottom part of Table 3, which gives the Pearson correlation matrix. The respective correlations of cases_per_10000 are: −0.3814 (*p*<0.01) with the second vaccination and −0.3921 (*p*<0.01) with the first vaccination.

### Methodology

Having demonstrated that the projected scope of COVID-19 morbidity *falls* with elevated vaccination rates for both doses separately, the next step forward would be to investigate the robustness of this prediction. The quadratic model permits non-monotonic variation, and the calculation of the vaccination rates, which yield the global minimum of COVID-19 scope of morbidity. Differently formulated, the global minimum represents the point where herd immunity is achieved within the city level.

Consider then the following quadratic models:

$$Cases\_per\_10,000=\alpha_{0}first\_vaccination\_sq+\alpha_{1}first\_vaccination+\alpha_{2}+\mu_{1}$$ (2)

$$Cases\_per\_10,000=\beta_{0}second\_vaccination\_sq+\beta_{1}second\_vaccination+\beta_{2}+\mu_{2}$$ (3)

Where *Cases*_*per*_10,000 is the dependent variable, *first*_*vaccination, first*_*vaccination*_*sq second*_*vaccination* and *second*_*vaccination*_*sq* are the independent variables, *first*_*vaccination*_*sq* = *first*_*vaccination*^2^, *second*_*vaccination*_*sq* = *second*_*vaccination*^2^, *α*_0_, *α*_1_, *α*_2_, *β*_0_, *β*_1_, *β*_2_ are parameters and *μ*_1_, *μ*_2_ are the stochastic random disturbance terms. It may be readily verified that: 1) a global minimum is achieved if ${\hat{\alpha }}_{0}>0$ and ${\hat{\beta }}_{0}>0$; 2) The vaccination rates that yield the minimum scope of COVID-19 morbidity are: $-\frac{{\hat{\alpha }}_{1}}{2{\hat{\alpha }}_{0}}$ and $-\frac{{\hat{\beta }}_{1}}{2{\hat{\beta }}_{0}}$; an 3) The minimum scope of morbidity is obtained by substitution of $-\frac{{\hat{\alpha }}_{1}}{2{\hat{\alpha }}_{0}}$ and $-\frac{{\hat{\beta }}_{1}}{2{\hat{\beta }}_{0}}$ in the respective estimated Eqs (2) and (3). (e.g., Chiang and Wainwright, 2005 [14]:226).

## Results

**Fig 4:**
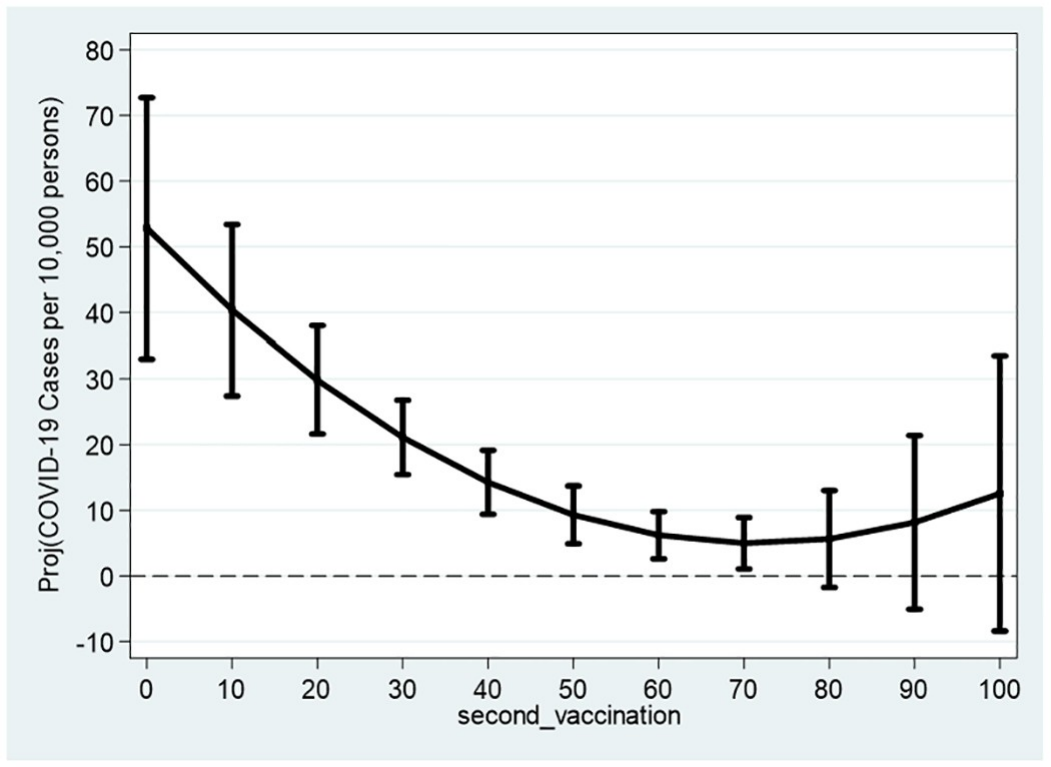
Second vaccination. Notes: The figures refer to 132 cities and towns and the estimation outcomes reported in Table 3.

**Fig 5:**
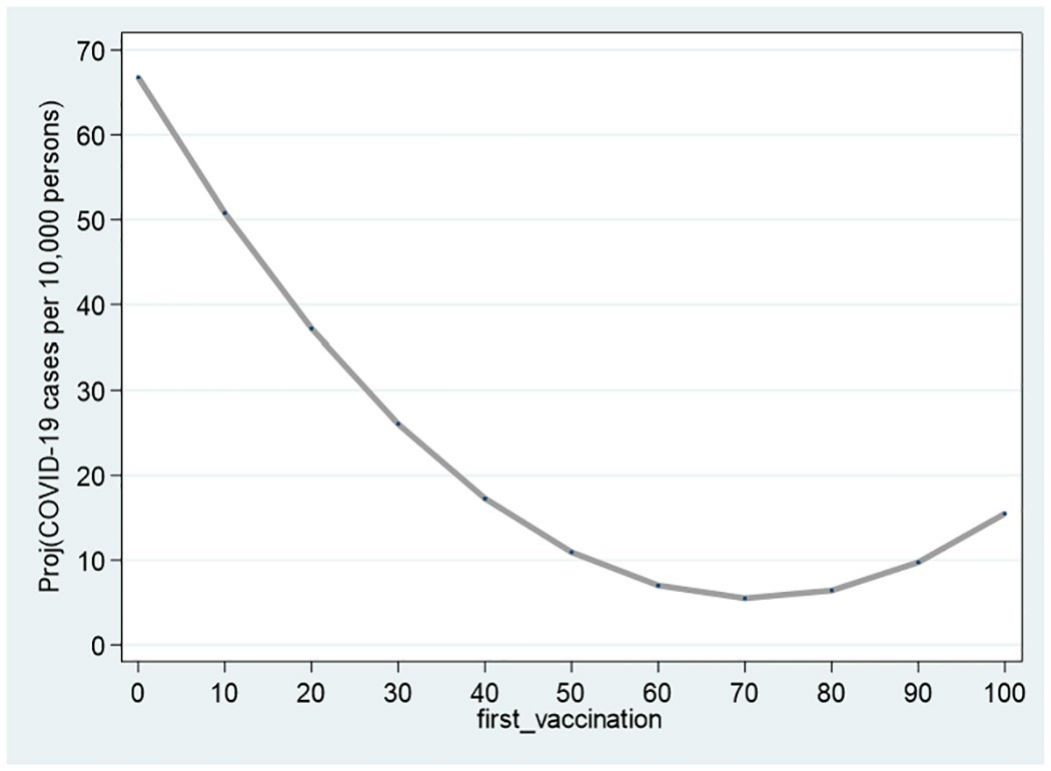
First vaccination. Notes: The figures refer to 132 cities and towns and the estimation outcomes reported in Table 3.

Table 4 reports the results obtained from the estimation of Eqs (2) and (3). For both models, the hypothesis that the quadratic model is more appropriate than the linear model to describe the data is supported empirically. The coefficients of second_vaccination_sq first_vaccination_sq are: 0.00936>0 (*p* = 0.0293) and 0.0121>0 (*p* = 0.00371), respectively. The fact that both parameters are positive also support the U-shaped curve with a global minimum for both the second and first vaccination. This is indeed demonstrated in Figs 4 and 5, respectively.

**Table 4:** Regression analysis.

VARIABLES		(1)	(2)	(3)	(4)
		cases_per_10,000	cases_per_10,000	cases_per_10,000	cases_per_10,000
Second_vaccination_sq	${\hat{\beta }}_{0}$	0.00936**	–	–	–
		(0.0293)	–	–	–
Second_vaccination	${\hat{\beta }}_{1}$	-1.339***	-0.430***	–	–
		(0.00189)	(6.41×10^−6^)	–	–
first_vaccination_sq	${\hat{\alpha }}_{0}$	–	–	0.0121***	–
		–	–	(0.00371)	–
first_vaccination	${\hat{\alpha }}_{1}$	–	–	-1.721***	-0.446***
		–	–	(0.000150)	(3.33×10^−6^)
Constant	${\hat{\beta }}_{2},{\hat{\alpha }}_{2}$	52.84***	34.12***	66.78***	37.87***
		(5.69×10^−7^)	(4.47×10^−9^)	(3.59×10^−8^)	(4.14×10^−9^)
Observations		132	132	132	132
R-squared		0.177	0.145	0.207	0.154
F(2,129)		13.83***	22.13***	16.88***	23.62***
Min. Projected Cases per 10,000 persons		52.84124 − 1.338662 × 71.52607 + 0.0093579 × 71.52607^2^ = 4.9668251		66.7815 − 1.720879 × 71.23255 + 0.0120793 × 71.23255^2^ = 5.490189	
Vaccination Rate (percent)	$-\frac{{\hat{\beta }}_{1}}{2{\hat{\beta }}_{0}},-\frac{{\hat{\alpha }}_{1}}{2{\hat{\alpha }}_{0}}$	$-\frac{\left(-1.339\right)}{2\times 0.00936}=71.52607$		$-\frac{\left(-1.720879\right)}{2\times 0.0120793}=71.23255$	
Projected cases per 10,000 persons for Vaccination Rate of 100%		52.84 − 1.339 × 100 + 0.00936 × 100^2^ = 12.54		66.78 − 1.721 × 100 + 0.0121 × 100^2^ = 15.68	

Notes: The table refers to 132 Israeli cities and towns, which cover above 5,964,295 inhabitants (above $\frac{5,964,295}{9,217,000}=64.71\%$ of the Israeli population). *P*-values are given in parentheses.

*p<0.1;

**p<0.05;

***p<0.01.

As these figures, as well as Table 3, illustrate, for cities with zero vaccination rates, the projected scopes of COVID-19 morbidity are: 52.84 (*p* = 5.69 × 10^−7^) and 66.78 (*p* = 3.59 × 10^−8^) active cases per 10,000 persons. These projected scopes of morbidity *fall* to a minimum of 4.966–5.49 active cases per 10,000 persons with elevation of vaccination rates to 71.12–71.53 percent of the city population. Above 71 percent vaccination rate, the projected scope of morbidity *rises* slightly to 12.54–15.68 active cases per 10,000 persons for a vaccination rate of 100 percent. Yet, the bottom part of Figs 2 and 3 show that based on the 95% confidence intervals, for the second (first) vaccination, above the threshold of 80 percent (90 percent), the null hypothesis of zero active cases per 10,000 persons cannot be rejected.

### Robustness test

One concern that should be addressed is reference to one source of immunity, namely, the percent of population who received the Pfizer vaccine. To address this concern we ran sensitivity tests referring to the second and first vaccinations. The outcomes of these tests are reported in Tables 5 and 6.

**Table 5:** Sensitivity analysis of the second vaccination where 0, 2, 4, 6, 8, 10 percent of the city population develops natural immunity.

VARIABLES		(1)	(2)	(3)	(4)	(5)	(6)
		cases_per_10000	cases_per_10000	cases_per_10000	cases_per_10000	cases_per_10000	cases_per_10000
Second_vaccination_sq	*a*	0.00936**	–	–	–	–	–
		(0.0293)	–	–	–	–	–
second_vaccination	*b*	-1.339***	–	–	–	–	–
		(0.00189)	–	–	–	–	–
(second_vaccination+2 percent)_sq	*a*	–	0.00936**	–	–	–	–
		–	(0.0293)	–	–	–	–
second_vaccination+2 percent	*b*	–	-1.376***	–	–	–	–
		–	(0.00211)	–	–	–	–
(second_vaccination+4 percent)_sq	*a*	–	–	0.00936**	–	–	–
		–	–	(0.0293)	–	–	–
second_vaccination+4 percent	*b*	–	–	-1.414***	–	–	–
		–	–	(0.00233)	–	–	–
(second_vaccination+6 percent)_sq	*a*	–	–	–	0.00936**	–	–
		–	–	–	(0.0293)	–	–
second_vaccination+6 percent	*b*	–	–	–	-1.451***	–	–
		–	–	–	(0.00257)	–	–
(second_vaccination+8 percent)_sq	*a*	–	–	–	–	0.00936**	–
		–	–	–	–	(0.0293)	–
second_vaccination+8 percent	*b*	–	–	–	–	-1.488***	–
		–	–	–	–	(0.00280)	–
(second_vaccination+10 percent)_sq	*a*	–	–	–	–	–	0.00936**
		–	–	–	–	–	(0.0293)
second_vaccination+10 percent	*b*	–	–	–	–	–	-1.526***
		–	–	–	–	–	(0.00304)
Constant	*c*	52.84***	55.56***	58.35***	61.21***	64.15***	67.16***
		(5.69×10^−7^)	(1.07×10^−6^)	(1.91×10^−6^)	(3.23×10^−6^)	(5.22×10^−6^)	(8.12×10^−6^)
Observations		132	132	132	132	132	132
R-squared		0.177	0.177	0.177	0.177	0.177	0.177
F-Statistics		13.83***	13.83***	13.83***	13.83***	13.83***	13.83***
Min. Projected Cases per 10,000 persons		4.9666	4.9666	4.9666	4.9666	4.9666	4.9666
95% confidence interval		[0.7211, 9.212]	[0.7211, 9.212]	[0.7211, 9.212]	[0.7211, 9.212]	[0.7211, 9.212]	[0.7211, 9.212]
Vaccinated in population (Min. Projected cases)	−*b*⁄2*a*	71.5261	73.5261	75.5261	77.5261	79.5261	81.5261

Notes: The Table refers to the second vaccination. The general form of the quadratic function is *f*(*x*) = *ax*^2^ + *bx* + *c* and a global minimum is obtained at $\frac{-b}{2a}$ where *a* > 0 (Chiang and Wainwright, 2005 [14]: 226). *P*-values are given in parentheses.

*p<0.1;

**p<0.05;

***p<0.01.

**Table 6:** Sensitivity analysis of the first vaccination where 0, 2, 4, 6, 8, 10 percent of the city population develops natural immunity.

VARIABLES		(1)	(2)	(3)	(4)	(5)	(6)
		cases_per_10000	cases_per_10000	cases_per_10000	cases_per_10000	cases_per_10000	cases_per_10000
first_vaccination_sq	*a*	0.0121***	–	–	–	–	–
		(0.0037)	–	–	–	–	–
first _vaccination	*b*	-1.7209***	–	–	–	–	–
		(0.0002)	–	–	–	–	–
(first _vaccination+2 percent)_sq	*a*	–	0.0121***	–	–	–	–
		–	(0.0037)	–	–	–	–
first_vaccination+2 percent	*b*	–	-1.7692***	–	–	–	–
		–	(0.0002)	–	–	–	–
(first _vaccination+4 percent)_sq	*a*	–	–	0.0121***	–	–	–
		–	–	(0.0037)	–	–	–
first_vaccination+4 percent	*b*	–	–	-1.8175***	–	–	–
		–	–	(0.0002)	–	–	–
(first_vaccination+6 percent)_sq	*a*	–	–	–	0.0121***	–	–
		–	–	–	(0.0037)	–	–
first_vaccination+6 percent	*b*	–	–	–	-1.8658***	–	–
		–	–	–	(0.0002)	–	–
(first_vaccination+8 percent)_sq	*a*	–	–	–	–	0.0121***	–
		–	–	–	–	(0.0037)	–
first_vaccination+8 percent	*b*	–	–	–	–	-1.9141***	–
		–	–	–	–	(0.0002)	–
(first_vaccination+10 percent)_sq	*a*	–	–	–	–	–	0.0121***
		–	–	–	–	–	(0.0037)
first_vaccination+10 percent	*b*	–	–	–	–	–	-1.9625***
		–	–	–	–	–	(0.0002)
Constant	*c*	66.7815***	70.2716***	73.8583***	77.5416***	81.3216***	85.1982***
		(<0.01)	(<0.01)	(<0.01)	(<0.01)	(<0.01)	(<0.01)
Observations		132	132	132	132	132	132
R-squared		0.2074	0.2074	0.2074	0.2074	0.2074	0.2074
F-Statistics		16.88***	16.88***	16.88***	16.88***	16.88***	16.88***
Min. Projected Cases per 10,000 persons		5.49019	5.49019	5.49019	5.49019	5.49019	5.49019
95% confidence interval		[2.1894, 8.7910]	[2.1894, 8.7910]	[2.1894, 8.7910]	[2.1894, 8.7910]	[2.1894, 8.7910]	[2.1894, 8.7910]
Vaccinated in population (Min. Projected cases)	−*b*⁄2*a*	71.23255	73.23255	75.23255	77.23255	79.23255	81.23255

Notes: The Table refers to the first vaccination. The general form of the quadratic function is *f*(*x*) = *ax*^2^ + *bx* + *c* and a global minimum is obtained at $\frac{-b}{2a}$ where *a* > 0. (Chiang and Wainwright, 2005 [14]: 226). *P*-values are given in parentheses.

*p<0.1;

**p<0.05;

***p<0.01.

Actually, the percentage of naturally immuned population is unknown. However, it is reasonable to consider candidates to naturally immuned persons from the group of unvaccinated persons. According to a 2021 US survey (Monte, L.M. [15])., the four dominant reasons people avoid any vaccinations are the following:

- Concern about side effects (49.6 percent)
- Mistrust of the vaccine (42.4 percent)
- Mistrust of the government (35.4 percent)
- Misbelief in the need of vaccine (31.8 percent)

In our sample, the median percent of persons who took the first dose of vaccine is 67.635, whereas in the united States the corresponding figure at that period is roughly 85 percent (e.g., Monte, L.M. [15]). The implication is that between 15 percent to 32.365 percent were not vaccinated at all. A reasonable assessment would be that approximately one-third of this group is naturally immuned.

Suppose then that 0, 2, 4, 6, 8, 10 percent of the city population develops natural immunity against SARS-Cov2 virus. We may define six independent variables where we supplemented to the second and first vaccination 0, 2, 4, 6, 8, 10 percent of naturally immuned populations. Results of this exercise is given in Tables 5 and 6– for the second and first vaccinations.

The outcomes remain robust regardless of the percent of the naturally immuned population. For the second vaccination, the minimum projected cases per 10,000 persons is 4.9666 persons obtained where the total percent of immuned persons (vaccinated + naturally immuned) is 71.5261, 73.5261, 75.5261, 77.5261, 79.5261, 81.5261.

For the first vaccination, the minimum projected cases per 10,000 persons is 5.49019 persons obtained where the total percent of immuned persons (vaccinated + naturally immuned) is 71.23255, 73.23255, 75.23255, 77.23255, 79.23255, 81.23255.

## Summary and conclusions

The objective of the current study is to propose a new approach to estimate the vaccination rates required to achieve herd immunity against SARS-COV2 virus at a city level. Based on information obtained from the Israeli Ministry of Health, we estimate two separate quadratic models, one for each dose of the BNT162b2 mRNA Pfizer vaccine. The dependent variable is the scope of morbidity, expressed as the number of cases per 10,000 persons. The independent variables are the first and second vaccination rates and their squares. Findings demonstrate that vaccination rate of 71 percent of the urban population is anticipated to yield the minimum scope of morbidity (approximately five COVID-19 cases per 10,000 persons).

High vaccination rates create virtual barriers to the spread of the pandemic, despite the lack of physical blockades for transportation from one city to another. Consequently, urban herd immunity may be defined as a situation where people continue to interact, yet the COVID-19 spread is not extended. This, in turn, would prevent the need for lockdowns or other limitations within the city level.

A potential limitation of the study is the implicit assumption according to which the only source of immunity against SARS-COV2 virus emanates from the COVID-19 Pfizer vaccinations. Consequently, according to one interpretation, the percent of vaccinated persons should be regarded as a lower bound for the extent of immuned population. Yet, this implicit assumption may be relaxed in the case that the unobserved percent of persons with a natural immunity is perfectly correlated with the percent of persons who are vaccinated.

To address this concern we ran a sensitivity analysis based on the assumption that 0, 2, 4, 6, 8, 10 percent of the city population develops natural immunity against SARS-CoV2 virus. Results remain robust to those obtained where none of the city population develops natural immunity.

## Supporting information

S1 Appendix Values of Herd Immunity Thresholds (HITs) of well-known infectious diseases. (DOCX)

S2 Appendix Reference list of S1 Appendix. (DOCX)

S1 File The raw data file. (XLSX)

S2 File The Stata do file. (DO)

S3 File The Stata log file. (PDF)
