# Supplementary material for: Can COVID-19 herd immunity be achieved at a city level?
Source: PLoS One. 2024 May 29;19(5):e0299574. doi: 10.1371/journal.pone.0299574 (PMC11135690; doi:10.1371/journal.pone.0299574)
Supplement: S1 Appendix — (DOCX) [file pone.0299574.s001.docx]

**Appendix**: Values of Herd Immunity Thresholds (HITs) of well-known infectious diseases

| **Disease** | **Transmission** | **HIT** | **References (1)** |
| --- | --- | --- | --- |
| Andes hantavirus | Respiratory droplets and body fluids | 16% (0–36%) | [1] |
| Chickenpox (varicella) | Aerosol | 90–92% | [2] |
| COVID-19 (Alpha variant) | Respiratory droplets and aerosol | 75–80% | [3] |
| COVID-19 (ancestral strain) | Respiratory droplets and aerosol [4] | 65% (58–71%) | [5] |
| COVID-19 (Delta variant) | Respiratory droplets and aerosol | 80% | [6] |
| Diphtheria | Saliva | 62% (41–77%) | [7] |
| Ebola (2014 outbreak) | Body fluids | 44% (31–44%) | [8],[9] |
| HIV/AIDS | Body fluids | 50–80% | [10] |
| Influenza (2009 pandemic strain) | Respiratory droplets | 37% (25–51%) | [11] |
| Influenza (seasonal strains) | Respiratory droplets | 23% (17–29%) | [12] |
| Measles | Aerosol | 92–94% | [13],[14] |
| Mumps | Respiratory droplets | 90–92% | [15] |
| Pertussis | Respiratory droplets | 82% | [16] |
| Polio | Fecal–oral route | 80–86% | [17],[18],[19] |
| Rubella | Respiratory droplets | 83–86% | [17],[18],[19] |
| SARS | Respiratory droplets | 50–75% | [20] |
| Smallpox | Respiratory droplets | 71–83% | [21],[22] |

1. See the reference list in Appendix S1.
