# Supplementary material for: Can COVID-19 herd immunity be achieved at a city level?
Source: PLoS One. 2024 May 29;19(5):e0299574. doi: 10.1371/journal.pone.0299574 (PMC11135690; doi:10.1371/journal.pone.0299574)
Supplement: S2 Appendix — (DOCX) [file pone.0299574.s002.docx]

**References**

## Martínez, Valeria P.; Di Paola, Nicholas; Alonso, Daniel O.; Pérez-Sautu, Unai; Bellomo, Carla M.; Iglesias, Ayelén A.; et al. (3 December 2020). "'Super-Spreaders' and Person-to-Person Transmission of Andes Virus in Argentina". New England Journal of Medicine. 383 (23): 2230–2241. [doi](https://en.wikipedia.org/wiki/Doi_(identifier)):[10.1056/NEJMoa2009040](https://doi.org/10.1056%2FNEJMoa2009040).

1. Ireland's Health Services. Health Care Worker Information*.* Available at: <https://www.hse.ie/eng/health/immunisation/hcpinfo/guidelines/chapter23.pdf> (last accessed January 11, 2022).
2. Gallagher, James (June 12, 2021). "Covid: Is there a limit to how much worse variants can get?". *BBC News*. Available at: <https://www.bbc.com/news/health-57431420> (last accessed October 7, 2023).
3. Prather, Kimberly A.; Marr, Linsey C.; Schooley, Robert T.; McDiarmid, Melissa A.; Wilson, Mary E.; Milton, Donald K. (16 October 2020). "Airborne transmission of SARS-CoV-2". *Science*. **370** (6514): 303.2–304.  [doi](https://en.wikipedia.org/wiki/Doi_(identifier)):[10.1126/science.abf0521](https://doi.org/10.1126%2Fscience.abf0521).
4. Billah, Arif; Miah, Mamun; Khan, Nuruzzaman (11 November 2020). "Reproductive number of coronavirus: A systematic review and meta-analysis based on global level evidence". *PLOS ONE*. 15 (11): e0242128.  [doi](https://en.wikipedia.org/wiki/Doi_(identifier)):[10.1371/journal.pone.0242128](https://doi.org/10.1371%2Fjournal.pone.0242128).
5. Liu, Ying; Rocklöv, Joacim (1 October 2021). "The reproductive number of the Delta variant of SARS-CoV-2 is far higher compared to the ancestral SARS-CoV-2 virus". *Journal of Travel Medicine*. **28** (7). [doi](https://en.wikipedia.org/wiki/Doi_(identifier)):[10.1093/jtm/taab124](https://doi.org/10.1093%2Fjtm%2Ftaab124).
6. Truelove SA, Keegan LT, Moss WJ, Chaisson LH, Macher E, Azman AS, Lessler J (June 2020). "Clinical and Epidemiological Aspects of Diphtheria: A Systematic Review and Pooled Analysis". *Clinical Infectious Diseases*. 71 (1): 89–97. [doi](https://en.wikipedia.org/wiki/Doi_(identifier)):[10.1093/cid/ciz808](https://doi.org/10.1093%2Fcid%2Fciz808).
7. Freeman C. (March. 2020) "Magic formula that will determine whether Ebola is beaten". *The Telegraph*. Telegraph.Co.Uk. Available at: <https://www.telegraph.co.uk/news/worldnews/ebola/11213280/Magic-formula-that-will-determine-whether-Ebola-is-beaten.html>.
8. Wong ZS, Bui CM, Chughtai AA, Macintyre CR (April 2017). "A systematic review of early modelling studies of Ebola virus disease in West Africa"*. Epidemiology and Infection.*145 (6): 1069–1094*.*[doi](https://en.wikipedia.org/wiki/Doi_(identifier)):[10.1017/S0950268817000164](https://doi.org/10.1017%2FS0950268817000164)
9. National Emerging Special Pathogen Training and Education Center (May 2020). *"Playing the Numbers Game: R0*"*..* Available at: <https://netec.org/2020/01/30/playing-the-numbers-game-r0/> (last accessed on January 5, 2022).
10. Fraser C, Donnelly CA, Cauchemez S, Hanage WP, Van Kerkhove MD, Hollingsworth TD, et al. (June 2009). "Pandemic potential of a strain of influenza A (H1N1): early findings". *Science*. **324** (5934): 1557–61. [doi](https://en.wikipedia.org/wiki/Doi_(identifier)):[10.1126/science.1176062](https://doi.org/10.1126%2Fscience.1176062).
11. Chowell G, Miller MA, Viboud C (June 2008). "Seasonal influenza in the United States, France, and Australia: transmission and prospects for control". *Epidemiology and Infection*. Cambridge University Press. **136** (6): 852–64. [doi](https://en.wikipedia.org/wiki/Doi_(identifier)):[10.1017/S0950268807009144](https://doi.org/10.1017%2FS0950268807009144).
12. Delamater PL, Street EJ, Leslie TF, Yang YT, Jacobsen KH (January 2019). "Complexity of the Basic Reproduction Number (R0)". *Emerging Infectious Diseases*. 25 (1): 1–4. [doi](https://en.wikipedia.org/wiki/Doi_(identifier)):[10.3201/eid2501.171901](https://doi.org/10.3201%2Feid2501.171901). [PMC](https://en.wikipedia.org/wiki/PMC_(identifier)) [6302597](https://www.ncbi.nlm.nih.gov/pmc/articles/PMC6302597). [PMID](https://en.wikipedia.org/wiki/PMID_(identifier)) [30560777](https://pubmed.ncbi.nlm.nih.gov/30560777)
13. Guerra FM, Bolotin S, Lim G, Heffernan J, Deeks SL, Li Y, Crowcroft NS (December 2017). "The basic reproduction number (R_0_) of measles: a systematic review". *The Lancet. Infectious Diseases*. 17 (12): e420–e428. [doi](https://en.wikipedia.org/wiki/Doi_(identifier)):[10.1016/S1473-3099(17)30307-9](https://doi.org/10.1016%2FS1473-3099%2817%2930307-9). [PMID](https://en.wikipedia.org/wiki/PMID_(identifier)) [28757186](https://pubmed.ncbi.nlm.nih.gov/28757186).
14. Australian government Department of Health Mumps Laboratory Case Definition (LCD). Available at: <https://www1.health.gov.au/internet/main/publishing.nsf/Content/cda-phlncd-mumps.htm>.
15. Kretzschmar M, Teunis PF, Pebody RG (June 2010). "Incidence and reproduction numbers of pertussis: estimates from serological and social contact data in five European countries". *PLOS Medicine*. **7** (6): e1000291. [doi](https://en.wikipedia.org/wiki/Doi_(identifier)):[10.1371/journal.pmed.1000291](https://doi.org/10.1371%2Fjournal.pmed.1000291).
16. Fine, Paul E. M. (1993). "Herd Immunity: History, Theory, Practice". *Epidemiologic Reviews*. **15** (2): 265–302. <https://doi.org/10.1093%2Foxfordjournals.epirev.a036121>.
17. Jiles, RB; Fuchs, C; Klevens, RM (22 September 2000). "Vaccination coverage among children enrolled in Head Start programs or day care facilities or entering school". *Morbidity and Mortality Weekly Report*. **49** (9): 27–38. PMID 11016876. Available at: <https://www.cdc.gov/mmwr/preview/mmwrhtml/ss4909a2.htm>.
18. Luman, ET; Barker, LE; Simpson, DM; Rodewald, LE; Szilagyi, PG; Zhao, Z (May 2001). "National, state, and urban-area vaccination-coverage levels among children aged 19–35 months, United States, 1999". *American Journal of Preventive Medicine*. **20** (4): 88–153. [doi](https://en.wikipedia.org/wiki/Doi_(identifier)):[10.1016/s0749-3797(01)00274-4](https://doi.org/10.1016%2Fs0749-3797%2801%2900274-4).
19. World Health Organization (2003). *Consensus document on the epidemiology of severe acute respiratory syndrome (SARS)*. Department of Communicable Disease Surveillance and Response (Technical report). . p. 26. [hdl](https://en.wikipedia.org/wiki/Hdl_(identifier)):[10665/70863](https://hdl.handle.net/10665%2F70863).
20. Centers for Disease Control and Prevention; World Health Organization (2001). "History and epidemiology of global smallpox eradication". Smallpox: disease, prevention, and intervention (training course). Available at: <https://stacks.cdc.gov/view/cdc/27929> (last accessed on January 11, 2022).
21. Gani R, Leach S (December 2001). "Transmission potential of smallpox in contemporary populations". *Nature*. **414** (6865): 748–51.  [doi](https://en.wikipedia.org/wiki/Doi_(identifier)):[10.1038/414748a](https://doi.org/10.1038%2F414748a).
