# Supplementary material for: Can COVID-19 herd immunity be achieved at a city level?
Source: PLoS One. 2024 May 29;19(5):e0299574. doi: 10.1371/journal.pone.0299574 (PMC11135690; doi:10.1371/journal.pone.0299574)
Supplement: S3 File — (PDF) [file pone.0299574.s005.pdf]

```
  ___  ____  ____  ____  ____(R)
 /__    /   ____/   /   ____/
___/   /   /___/   /   /___/
  Statistics/Data analysis

    User: Replication
 Project: Herd immunity
```

```
      name:  herd_immunity
       log:  C:\My Passport\papers_netanya\Lamas_datasets_via_huji\Vaccinated_by_Yishuv_20210922\Fifth_Paper\PLOS_ON
  log type:  smcl
 opened on:   6 Oct 2023, 17:09:12

1 . set more off

2 . cd "C:\My Passport\papers_netanya\Lamas_datasets_via_huji\Vaccinated_by_Yishuv_20210922\Fifth_Paper"
  C:\My Passport\papers_netanya\Lamas_datasets_via_huji\Vaccinated_by_Yishuv_20210922\Fifth_Paper

3 . import excel "vaccination_by_settlements.xlsx", sheet("vaccination_by_settlements") firstrow clear
  (8 vars, 280 obs)

4 . ///Table 1: Descriptive Statistics
  > gen diff_first_second=second_vaccination-first_vaccination

5 . summ cases_per_10000 second_vaccination first_vaccination diff_first_second if cases_per_10000!=.
```

Variable	Obs	Mean	Std. Dev.	Min	Max
cases_~10000	132	9.656061	19.15019	0	135.4
second_vac~n	132	56.86735	16.98323	6.83	90
first_vacc~n	132	63.26886	16.84056	8.32	90
diff_first~d	132	-6.401515	2.543937	-15.74	0

```
6 . ttest cases_per_10000=0, level(99)

  One-sample t test
```

Variable	Obs	Mean	Std. Err.	Std. Dev.	[99% Conf.	Interval]
ca~10000	132	9.656061	1.66681	19.15019	5.299218	14.0129

```
    mean = mean(cases_per_10000)                                  t =   5.7931
Ho: mean = 0                                     degrees of freedom =      131

    Ha: mean < 0                 Ha: mean != 0                 Ha: mean > 0
 Pr(T < t) = 1.0000         Pr(|T| > |t|) = 0.0000          Pr(T > t) = 0.0000

7 . ttest second_vaccination==0 if cases_per_10000!=.

  One-sample t test
```

Variable	Obs	Mean	Std. Err.	Std. Dev.	[95% Conf.	Interval]
second~n	132	56.86735	1.4782	16.98323	53.94312	59.79158

```
    mean = mean(second_vaccination)                               t =  38.4707
Ho: mean = 0                                     degrees of freedom =      131

    Ha: mean < 0                 Ha: mean != 0                 Ha: mean > 0
 Pr(T < t) = 1.0000         Pr(|T| > |t|) = 0.0000          Pr(T > t) = 0.0000
```

8 . ttest first_vaccination==0 if cases_per_10000!=.

One-sample t test

Variable	Obs	Mean	Std. Err.	Std. Dev.	[95% Conf.	Interval]
first_~n	**132**	**63.26886**	**1.465783**	**16.84056**	**60.3692**	**66.16853**

mean = mean(**first_vaccination**)　　t = **43.1639**
Ho: mean = **0**　　degrees of freedom = **131**

Ha: mean < **0**	Ha: mean != **0**	Ha: mean > **0**
Pr(T < t) = **1.0000**	Pr(|T| > |t|) = **0.0000**	Pr(T > t) = **0.0000**

9 . ttest diff_first_second==0 if cases_per_10000!=.

One-sample t test

Variable	Obs	Mean	Std. Err.	Std. Dev.	[95% Conf.	Interval]
diff_f~d	**132**	**-6.401515**	**.2214213**	**2.543937**	**-6.839539**	**-5.963491**

mean = mean(**diff_first_second**)　　t = **-28.9110**
Ho: mean = **0**　　degrees of freedom = **131**

Ha: mean < **0**	Ha: mean != **0**	Ha: mean > **0**
Pr(T < t) = **0.0000**	Pr(|T| > |t|) = **0.0000**	Pr(T > t) = **1.0000**

10 . //Figure 1: Distribution of COVID-19 Cases per 10,000 persons
11 . histogram cases_per_10000, percent width(10) addlabels
(bin=**14**, start=**0**, width=**10**)

12 . //Figure 2: Distribution of Percent of Vaccinated Persons
13 . histogram first_vaccination if cases_per_10000!=., percent width(10) addlabels
(bin=**9**, start=**8.32**, width=**10**)

14 . histogram second_vaccination if cases_per_10000!=., percent width(10) addlabels
(bin=**9**, start=**6.83**, width=**10**)

15 . //Table 2: Collinearity and Pearson Correlation Matrix
16 . //Table 2: A. Regression Analysis (132 Cities and Towns)
17 . reg cases_per_10000 second_vaccination first_vaccination

Source	SS	df	MS			
				Number of obs	=	**132**
				F(2, 129)	=	**11.87**
Model	**7469.28171**	**2**	**3734.64085**	Prob > F	=	**0.0000**
Residual	**40572.3234**	**129**	**314.514135**	R-squared	=	**0.1555**
				Adj R-squared	=	**0.1424**
Total	**48041.6052**	**131**	**366.72981**	Root MSE	=	**17.735**

cases_per_10000	Coef.	Std. Err.	t	P>|t|	[95% Conf.	Interval]
second_vaccination	**.3135739**	**.6091977**	**0.51**	**0.608**	**-.8917388**	**1.518887**
first_vaccination	**-.7585366**	**.6143586**	**-1.23**	**0.219**	**-1.97406**	**.4569868**
_cons	**39.8157**	**7.114654**	**5.60**	**0.000**	**25.73918**	**53.89221**

```
18 . outreg2 using Table2A.xls,replace pval
   Table2A.xls
   dir : seeout

19 . reg cases_per_10000 second_vaccination

         Source |       SS           df       MS      Number of obs   =       132
   -------------+----------------------------------   F(1, 130)       =     22.13
          Model |  6989.82494         1  6989.82494   Prob > F        =    0.0000
       Residual |  41051.7802       130  315.782925   R-squared       =    0.1455
   -------------+----------------------------------   Adj R-squared   =    0.1389
          Total |  48041.6052       131   366.72981   Root MSE        =     17.77

   ------------------------------------------------------------------------------------
      cases_per_10000 |      Coef.   Std. Err.      t    P>|t|     [95% Conf. Interval]
   -------------------+----------------------------------------------------------------
   second_vaccination |  -.4301078   .0914194    -4.70   0.000    -.6109702   -.2492454
                _cons |   34.11515   5.423985     6.29   0.000     23.38444    44.84585
   ------------------------------------------------------------------------------------

20 . outreg2 using Table2A.xls, pval
   Table2A.xls
   dir : seeout

21 . reg cases_per_10000 first_vaccination

         Source |       SS           df       MS      Number of obs   =       132
   -------------+----------------------------------   F(1, 130)       =     23.62
          Model |  7385.95134         1  7385.95134   Prob > F        =    0.0000
       Residual |  40655.6538       130  312.735799   R-squared       =    0.1537
   -------------+----------------------------------   Adj R-squared   =    0.1472
          Total |  48041.6052       131   366.72981   Root MSE        =    17.684

   -----------------------------------------------------------------------------------
     cases_per_10000 |      Coef.   Std. Err.      t    P>|t|     [95% Conf. Interval]
   ------------------+----------------------------------------------------------------
   first_vaccination |  -.4458728    .091748    -4.86   0.000    -.6273853   -.2643604
               _cons |   37.86593   6.005399     6.31   0.000     25.98496    49.74689
   -----------------------------------------------------------------------------------

22 . outreg2 using Table2A.xls, pval
   Table2A.xls
   dir : seeout

23 . //Table 2: B. Correlation Matrix (132 Cities and Towns)
24 . pwcorr cases_per_10000 second_vaccination first_vaccination if cases_per_10000!=., sig obs star(0.01)

                |  ca~10000 second~n first_~n
   -------------+---------------------------
   cases_~10000 |   1.0000
                |
                |      132
                |
   second_vac~n |  -0.3814*  1.0000
                |   0.0000
                |      132      132
                |
   first_vacc~n |  -0.3921*  0.9887*  1.0000
                |   0.0000   0.0000
                |      132      132      132
                |
```

```
25 . //Table 3: Regression Analysis
26 . reg cases_per_10000 c.second_vaccination##c.second_vaccination

      Source |       SS           df       MS      Number of obs   =       132
-------------+----------------------------------   F(2, 129)       =     13.83
       Model |  8480.12219         2  4240.0611   Prob > F        =    0.0000
    Residual |   39561.483       129  306.678162   R-squared       =    0.1765
-------------+----------------------------------   Adj R-squared   =    0.1637
       Total |  48041.6052       131   366.72981   Root MSE        =    17.512

------------------------------------------------------------------------------------------------------------
                                cases_per_10000 |      Coef.   Std. Err.      t    P>|t|     [95% Conf. Interval]
------------------------------------------------+-----------------------------------------------------------
                             second_vaccination |  -1.338662   .4218822    -3.17   0.002    -2.173366   -.5039578
                                                |
c.second_vaccination#c.second_vaccination       |   .0093579    .004245     2.20   0.029     .0009589    .0177568
                                                |
                                          _cons |   52.84124   10.03657     5.26   0.000     32.98365    72.69883
------------------------------------------------------------------------------------------------------------

27 . outreg2 using Table3.xls,replace pval
   Table3.xls
   dir : seeout

28 . reg cases_per_10000 second_vaccination

      Source |       SS           df       MS      Number of obs   =       132
-------------+----------------------------------   F(1, 130)       =     22.13
       Model |  6989.82494         1  6989.82494   Prob > F        =    0.0000
    Residual |  41051.7802       130  315.782925   R-squared       =    0.1455
-------------+----------------------------------   Adj R-squared   =    0.1389
       Total |  48041.6052       131   366.72981   Root MSE        =     17.77

------------------------------------------------------------------------------------
   cases_per_10000 |      Coef.   Std. Err.      t    P>|t|     [95% Conf. Interval]
-------------------+----------------------------------------------------------------
second_vaccination |  -.4301078   .0914194    -4.70   0.000    -.6109702   -.2492454
             _cons |   34.11515   5.423985     6.29   0.000     23.38444    44.84585
------------------------------------------------------------------------------------

29 . outreg2 using Table3.xls,pval
   Table3.xls
   dir : seeout

30 . reg cases_per_10000 c.first_vaccination##c.first_vaccination

      Source |       SS           df       MS      Number of obs   =       132
-------------+----------------------------------   F(2, 129)       =     16.88
       Model |  9964.96241         2  4982.48121   Prob > F        =    0.0000
    Residual |  38076.6427       129  295.167773   R-squared       =    0.2074
-------------+----------------------------------   Adj R-squared   =    0.1951
       Total |  48041.6052       131   366.72981   Root MSE        =     17.18

----------------------------------------------------------------------------------------------------------
                              cases_per_10000 |      Coef.   Std. Err.      t    P>|t|     [95% Conf. Interval]
----------------------------------------------+-----------------------------------------------------------
                            first_vaccination |  -1.720879   .4404537    -3.91   0.000    -2.592328   -.8494308
                                              |
c.first_vaccination#c.first_vaccination       |   .0120793   .0040865     2.96   0.004     .0039941    .0201645
                                              |
                                        _cons |    66.7815   11.38998     5.86   0.000     44.24614    89.31686
----------------------------------------------------------------------------------------------------------
```

```
31 . outreg2 using Table3.xls, pval
   Table3.xls
   dir : seeout

32 . reg cases_per_10000 first_vaccination

         Source |       SS           df       MS      Number of obs   =       132
   -------------+----------------------------------   F(1, 130)       =     23.62
          Model |  7385.95134         1  7385.95134   Prob > F        =    0.0000
       Residual |  40655.6538       130  312.735799   R-squared       =    0.1537
   -------------+----------------------------------   Adj R-squared   =    0.1472
          Total |  48041.6052       131   366.72981   Root MSE        =    17.684

   ------------------------------------------------------------------------------------
     cases_per_10000 |      Coef.   Std. Err.      t    P>|t|     [95% Conf. Interval]
   ------------------+-----------------------------------------------------------------
   first_vaccination |  -.4458728    .091748    -4.86   0.000    -.6273853   -.2643604
               _cons |   37.86593   6.005399     6.31   0.000     25.98496    49.74689
   ------------------------------------------------------------------------------------

33 . outreg2 using Table3.xls, pval
   Table3.xls
   dir : seeout

34 . //Figure 3: Second Vaccination
35 . reg cases_per_10000 c.second_vaccination##c.second_vaccination

         Source |       SS           df       MS      Number of obs   =       132
   -------------+----------------------------------   F(2, 129)       =     13.83
          Model |  8480.12219         2   4240.0611   Prob > F        =    0.0000
       Residual |   39561.483       129  306.678162   R-squared       =    0.1765
   -------------+----------------------------------   Adj R-squared   =    0.1637
          Total |  48041.6052       131   366.72981   Root MSE        =    17.512

   --------------------------------------------------------------------------------------------------------------
                               cases_per_10000 |      Coef.   Std. Err.      t    P>|t|     [95% Conf. Interval]
   --------------------------------------------+-----------------------------------------------------------------
                            second_vaccination |  -1.338662   .4218822    -3.17   0.002    -2.173366   -.5039578
                                               |
   c.second_vaccination#c.second_vaccination   |   .0093579    .004245     2.20   0.029     .0009589    .0177568
                                               |
                                         _cons |   52.84124   10.03657     5.26   0.000     32.98365    72.69883
   --------------------------------------------------------------------------------------------------------------

36 . margins, at(second_vaccination==(0(10)100))

   Adjusted predictions                            Number of obs     =        132
   Model VCE    : OLS

   Expression   : Linear prediction, predict()

   1._at        : second_vac~n    =           0

   2._at        : second_vac~n    =          10

   3._at        : second_vac~n    =          20

   4._at        : second_vac~n    =          30

   5._at        : second_vac~n    =          40

   6._at        : second_vac~n    =          50

   7._at        : second_vac~n    =          60
```

```
8._at        : second_vac~n    =          70

9._at        : second_vac~n    =          80

10._at       : second_vac~n    =          90

11._at       : second_vac~n    =         100
```

	Margin	Delta-method Std. Err.	t	P>|t|	[95% Conf.	Interval]
_at						
1	52.84124	10.03657	5.26	0.000	32.98365	72.69883
2	40.39041	6.570416	6.15	0.000	27.39068	53.39013
3	29.81114	4.1421	7.20	0.000	21.6159	38.00639
4	21.10345	2.860892	7.38	0.000	15.44311	26.7638
5	14.26734	2.463853	5.79	0.000	9.392541	19.14213
6	9.302789	2.226333	4.18	0.000	4.897936	13.70764
7	6.209816	1.819202	3.41	0.001	2.61048	9.809152
8	4.988414	1.98018	2.52	0.013	1.07058	8.906248
9	5.638585	3.729344	1.51	0.133	-1.740015	13.01718
10	8.160327	6.686679	1.22	0.225	-5.069431	21.39009
11	12.55364	10.58766	1.19	0.238	-8.394296	33.50158

37 . marginsplot, noci

Variables that uniquely identify margins: second_vaccination

38 . marginsplot

Variables that uniquely identify margins: second_vaccination

39 . reg cases_per_10000 c.first_vaccination##c.first_vaccination

Source	SS	df	MS
Model	9964.96241	2	4982.48121
Residual	38076.6427	129	295.167773
Total	48041.6052	131	366.72981

Number of obs	=	132
F(2, 129)	=	16.88
Prob > F	=	0.0000
R-squared	=	0.2074
Adj R-squared	=	0.1951
Root MSE	=	17.18

cases_per_10000	Coef.	Std. Err.	t	P>|t|	[95% Conf.	Interval]
first_vaccination	-1.720879	.4404537	-3.91	0.000	-2.592328	-.8494308
c.first_vaccination#c.first_vaccination	.0120793	.0040865	2.96	0.004	.0039941	.0201645
_cons	66.7815	11.38998	5.86	0.000	44.24614	89.31686

```
40 . margins, at(first_vaccination==(0(10)100))

   Adjusted predictions                              Number of obs     =        132
   Model VCE    : OLS

   Expression   : Linear prediction, predict()

   1._at        : first_vacc~n    =           0

   2._at        : first_vacc~n    =          10

   3._at        : first_vacc~n    =          20

   4._at        : first_vacc~n    =          30

   5._at        : first_vacc~n    =          40

   6._at        : first_vacc~n    =          50

   7._at        : first_vacc~n    =          60

   8._at        : first_vacc~n    =          70

   9._at        : first_vacc~n    =          80

   10._at       : first_vacc~n    =          90

   11._at       : first_vacc~n    =         100
```

	Margin	Delta-method Std. Err.	t	P>|t|	[95% Conf.	Interval]
_at						
1	66.7815	11.38998	5.86	0.000	44.24614	89.31686
2	50.78064	7.702293	6.59	0.000	35.54147	66.01982
3	37.19564	4.989468	7.45	0.000	27.32385	47.06742
4	26.0265	3.361529	7.74	0.000	19.37563	32.67737
5	17.27322	2.721803	6.35	0.000	11.88807	22.65837
6	10.9358	2.468854	4.43	0.000	6.051113	15.82049
7	7.014243	2.060164	3.40	0.001	2.938158	11.09033
8	5.508546	1.657247	3.32	0.001	2.229641	8.78745
9	6.41871	2.549669	2.52	0.013	1.374127	11.46329
10	9.744735	4.941068	1.97	0.051	-.0312885	19.52076
11	15.48662	8.330736	1.86	0.065	-.9959441	31.96919

```
41 . marginsplot, noci

     Variables that uniquely identify margins: first_vaccination

42 . marginsplot

     Variables that uniquely identify margins: first_vaccination
```

```
43 . //Table 5: Sensitivity Analysis of the Second Vaccination
44 . //where 0, 2, 4, 6, 8, 10 percent of the city population develops natural Immunity.
45 . gen second_vaccination2=second_vaccination+2

46 . gen second_vaccination4=second_vaccination+4

47 . gen second_vaccination6=second_vaccination+6

48 . gen second_vaccination8=second_vaccination+8

49 . gen second_vaccination10=second_vaccination+10

50 . gen second_vaccination_sq=second_vaccination*second_vaccination

51 . gen second_vaccination2_sq=second_vaccination2*second_vaccination2

52 . gen second_vaccination4_sq=second_vaccination4*second_vaccination4

53 . gen second_vaccination6_sq=second_vaccination6*second_vaccination6

54 . gen second_vaccination8_sq=second_vaccination8*second_vaccination8

55 . gen second_vaccination10_sq=second_vaccination10*second_vaccination10

56 . reg cases_per_10000 c.second_vaccination##c.second_vaccination
```

Source	SS	df	MS			
Model	**8480.12219**	**2**	**4240.0611**	Number of obs	=	**132**
Residual	**39561.483**	**129**	**306.678162**	F(2, 129)	=	**13.83**
				Prob > F	=	**0.0000**
				R-squared	=	**0.1765**
				Adj R-squared	=	**0.1637**
Total	**48041.6052**	**131**	**366.72981**	Root MSE	=	**17.512**

cases_per_10000	Coef.	Std. Err.	t	P>|t|	[95% Conf.	Interval]
second_vaccination	**-1.338662**	**.4218822**	**-3.17**	**0.002**	**-2.173366**	**-.5039578**
c.second_vaccination#c.second_vaccination	**.0093579**	**.004245**	**2.20**	**0.029**	**.0009589**	**.0177568**
_cons	**52.84124**	**10.03657**	**5.26**	**0.000**	**32.98365**	**72.69883**

```
57 . outreg2 using Table4.xls,replace pval addstat(F-statistics, e(F))
   Table4.xls
   dir : seeout

58 . nlcom -_b[second_vaccination]/(2*_b[c.second_vaccination#c.second_vaccination])

       _nl_1:  -_b[second_vaccination]/(2*_b[c.second_vaccination#c.second_vaccination])
```

cases_~10000	Coef.	Std. Err.	z	P>|z|	[95% Conf.	Interval]
_nl_1	**71.52607**	**11.48269**	**6.23**	**0.000**	**49.02042**	**94.03173**

```
59 . reg cases_per_10000 second_vaccination second_vaccination_sq
```

Source	SS	df	MS
Model	8480.12197	2	4240.06099
Residual	39561.4832	129	306.678164
Total	48041.6052	131	366.72981

Number of obs	=	132
F(2, 129)	=	13.83
Prob > F	=	0.0000
R-squared	=	0.1765
Adj R-squared	=	0.1637
Root MSE	=	17.512

cases_per_10000	Coef.	Std. Err.	t	P>|t|	[95% Conf.	Interval]
second_vaccination	-1.338662	.4218822	-3.17	0.002	-2.173366	-.5039577
second_vaccination_sq	.0093579	.004245	2.20	0.029	.0009589	.0177568
_cons	52.84124	10.03657	5.26	0.000	32.98365	72.69883

```
60 . quietly display  52.84124-1.338662*71.52607+.0093579*(71.52607^2)  //4.9668251

61 . margins, at(second_vaccination== 71.52607 second_vaccination_sq==5115.9787)
```

Adjusted predictions   Number of obs = 132
Model VCE   : **OLS**

Expression   : **Linear prediction, predict()**
at   : second_vac~n   =   **71.52607**
               second_~n_sq   =   **5115.979**

	Delta-method					
	Margin	Std. Err.	t	P>|t|	[95% Conf.	Interval]
_cons	4.966619	2.145781	2.31	0.022	.7211397	9.212099

```
62 . reg cases_per_10000 c.second_vaccination2##c.second_vaccination2
```

Source	SS	df	MS
Model	8480.12214	2	4240.06107
Residual	39561.483	129	306.678163
Total	48041.6052	131	366.72981

Number of obs	=	132
F(2, 129)	=	13.83
Prob > F	=	0.0000
R-squared	=	0.1765
Adj R-squared	=	0.1637
Root MSE	=	17.512

cases_per_10000	Coef.	Std. Err.	t	P>|t|	[95% Conf.	Interval]
second_vaccination2	-1.376093	.4384857	-3.14	0.002	-2.243648	-.5085388
c.second_vaccination2#c.second_vaccination2	.0093579	.004245	2.20	0.029	.0009589	.0177568
_cons	55.55599	10.84491	5.12	0.000	34.09907	77.01292

```
63 . nlcom -_b[second_vaccination2]/(2*_b[c.second_vaccination2#c.second_vaccination2])

       _nl_1:  -_b[second_vaccination2]/(2*_b[c.second_vaccination2#c.second_vaccination2])

------------------------------------------------------------------------------
cases_~10000 |      Coef.   Std. Err.      z    P>|z|     [95% Conf. Interval]
-------------+----------------------------------------------------------------
       _nl_1 |   73.52607   11.48269     6.40   0.000     51.02041    96.03173
------------------------------------------------------------------------------

64 . outreg2 using Table4.xls, pval addstat(F-statistics, e(F))
   Table4.xls
   dir : seeout

65 . quietly display 73.52607^2  //5406.083

66 . reg cases_per_10000 second_vaccination2 second_vaccination2_sq

      Source |       SS           df       MS      Number of obs   =       132
-------------+----------------------------------   F(2, 129)       =     13.83
       Model |  8480.12241         2  4240.0612    Prob > F        =    0.0000
    Residual |  39561.4827       129  306.678161   R-squared       =    0.1765
-------------+----------------------------------   Adj R-squared   =    0.1637
       Total |  48041.6052       131   366.72981   Root MSE        =    17.512

-------------------------------------------------------------------------------------
    cases_per_10000 |      Coef.   Std. Err.      t    P>|t|     [95% Conf. Interval]
--------------------+----------------------------------------------------------------
second_vaccination2 |  -1.376094   .4384857    -3.14   0.002    -2.243648   -.5085389
second_vaccination2_sq |   .0093579    .004245     2.20   0.029     .0009589    .0177568
              _cons |     55.556   10.84491     5.12   0.000     34.09907    77.01292
-------------------------------------------------------------------------------------

67 . margins, at(second_vaccination2== 73.52607 second_vaccination2_sq==5406.083)

   Adjusted predictions                            Number of obs     =        132
   Model VCE    : OLS

   Expression   : Linear prediction, predict()
   at           : second_vac~2    =    73.52607
                  second_~2_sq    =    5406.083

------------------------------------------------------------------------------
             |            Delta-method
             |     Margin   Std. Err.      t    P>|t|     [95% Conf. Interval]
-------------+----------------------------------------------------------------
       _cons |   4.966621   2.145781     2.31   0.022     .7211412    9.212102
------------------------------------------------------------------------------

68 . reg cases_per_10000 c.second_vaccination4##c.second_vaccination4

      Source |       SS           df       MS      Number of obs   =       132
-------------+----------------------------------   F(2, 129)       =     13.83
       Model |  8480.12205         2  4240.06102   Prob > F        =    0.0000
    Residual |  39561.4831       129  306.678164   R-squared       =    0.1765
-------------+----------------------------------   Adj R-squared   =    0.1637
       Total |  48041.6052       131   366.72981   Root MSE        =    17.512
```

cases_per_10000	Coef.	Std. Err.	t	P>|t|	[95% Conf.	Interval]
second_vaccination4	**-1.413525**	**.4551169**	**-3.11**	**0.002**	**-2.313985**	**-.5130649**
c.second_vaccination4#c.second_vaccination4	**.0093579**	**.004245**	**2.20**	**0.029**	**.0009589**	**.0177568**
_cons	**58.34561**	**11.69036**	**4.99**	**0.000**	**35.21595**	**81.47527**

69 . nlcom -_b[second_vaccination4]/(2*_b[c.second_vaccination#c.second_vaccination4])

_nl_1: **-_b[second_vaccination4]/(2*_b[c.second_vaccination#c.second_vaccination4])**

cases_~10000	Coef.	Std. Err.	z	P>|z|	[95% Conf.	Interval]
_nl_1	**75.52607**	**11.48269**	**6.58**	**0.000**	**53.02041**	**98.03173**

70 . outreg2 using Table4.xls, pval addstat(F-statistics, e(F))
Table4.xls
dir : seeout

71 . quietly display 75.52607^2 //5704.1872

72 . reg cases_per_10000 second_vaccination4 second_vaccination4_sq

Source	SS	df	MS
Model	**8480.12231**	**2**	**4240.06115**
Residual	**39561.4828**	**129**	**306.678162**
Total	**48041.6052**	**131**	**366.72981**

Number of obs	=	**132**
F(2, 129)	=	**13.83**
Prob > F	=	**0.0000**
R-squared	=	**0.1765**
Adj R-squared	=	**0.1637**
Root MSE	=	**17.512**

cases_per_10000	Coef.	Std. Err.	t	P>|t|	[95% Conf.	Interval]
second_vaccination4	**-1.413525**	**.4551169**	**-3.11**	**0.002**	**-2.313985**	**-.513065**
second_vaccination4_sq	**.0093579**	**.004245**	**2.20**	**0.029**	**.0009589**	**.0177568**
_cons	**58.34561**	**11.69036**	**4.99**	**0.000**	**35.21596**	**81.47527**

73 . margins, at(second_vaccination4== 75.52607 second_vaccination4_sq==5704.1872)

Adjusted predictions   Number of obs = **132**
Model VCE   : **OLS**

Expression   : **Linear prediction, predict()**
at   : second_vac~4   =   **75.52607**
               second_~4_sq   =   **5704.187**

	Margin	Delta-method Std. Err.	t	P>|t|	[95% Conf.	Interval]
_cons	**4.966619**	**2.145781**	**2.31**	**0.022**	**.7211394**	**9.212098**

```
74 . reg cases_per_10000 c.second_vaccination6##c.second_vaccination6

      Source |       SS           df       MS      Number of obs   =       132
-------------+----------------------------------   F(2, 129)       =     13.83
       Model |  8480.12205         2  4240.06102   Prob > F        =    0.0000
    Residual |  39561.4831       129  306.678164   R-squared       =    0.1765
-------------+----------------------------------   Adj R-squared   =    0.1637
       Total |  48041.6052       131   366.72981   Root MSE        =    17.512

------------------------------------------------------------------------------------------------------------
                                cases_per_10000 |      Coef.   Std. Err.      t    P>|t|     [95% Conf. Interval]
------------------------------------------------+-----------------------------------------------------------
                            second_vaccination6 |  -1.450956    .471773    -3.08   0.003    -2.384371   -.5175418
                                                |
c.second_vaccination6#c.second_vaccination6 |   .0093579    .004245     2.20   0.029     .0009589    .0177568
                                                |
                                          _cons |   61.21009   12.57251     4.87   0.000     36.33508    86.08511
------------------------------------------------------------------------------------------------------------

75 . nlcom -_b[second_vaccination6]/(2*_b[c.second_vaccination6#c.second_vaccination6])

       _nl_1:  -_b[second_vaccination6]/(2*_b[c.second_vaccination6#c.second_vaccination6])

------------------------------------------------------------------------------
cases_~10000 |      Coef.   Std. Err.      z    P>|z|     [95% Conf. Interval]
-------------+----------------------------------------------------------------
       _nl_1 |   77.52607   11.48269     6.75   0.000     55.02041    100.0317
------------------------------------------------------------------------------

76 . outreg2 using Table4.xls, pval addstat(F-statistics, e(F))
   Table4.xls
   dir : seeout

77 . quietly display 77.52607^2  //6010.2915

78 . reg cases_per_10000 second_vaccination6 second_vaccination6_sq

      Source |       SS           df       MS      Number of obs   =       132
-------------+----------------------------------   F(2, 129)       =     13.83
       Model |  8480.12199         2  4240.06099   Prob > F        =    0.0000
    Residual |  39561.4832       129  306.678164   R-squared       =    0.1765
-------------+----------------------------------   Adj R-squared   =    0.1637
       Total |  48041.6052       131   366.72981   Root MSE        =    17.512

----------------------------------------------------------------------------------------
       cases_per_10000 |      Coef.   Std. Err.      t    P>|t|     [95% Conf. Interval]
-----------------------+----------------------------------------------------------------
   second_vaccination6 |  -1.450956    .471773    -3.08   0.003    -2.384371   -.5175418
second_vaccination6_sq |   .0093579    .004245     2.20   0.029     .0009589    .0177568
                 _cons |   61.21009   12.57251     4.87   0.000     36.33508    86.08511
----------------------------------------------------------------------------------------

79 . margins, at(second_vaccination6== 77.52607 second_vaccination6_sq==6010.2915)

Adjusted predictions                            Number of obs     =        132
Model VCE    : OLS

Expression   : Linear prediction, predict()
at           : second_vac~6    =    77.52607
               second_~6_sq    =    6010.292
```

```
------------------------------------------------------------------------------
             |            Delta-method
             |     Margin   Std. Err.      t    P>|t|     [95% Conf. Interval]
-------------+----------------------------------------------------------------
       _cons |   4.966621   2.145781     2.31   0.022     .7211407    9.212101
------------------------------------------------------------------------------

80 . reg cases_per_10000 c.second_vaccination8##c.second_vaccination8

      Source |       SS           df       MS      Number of obs   =       132
-------------+----------------------------------   F(2, 129)       =     13.83
       Model |  8480.12202         2  4240.06101   Prob > F        =    0.0000
    Residual |  39561.4831       129  306.678164   R-squared       =    0.1765
-------------+----------------------------------   Adj R-squared   =    0.1637
       Total |  48041.6052       131   366.72981   Root MSE        =    17.512

---------------------------------------------------------------------------------------------------------------
                                      cases_per_10000 |      Coef.   Std. Err.      t    P>|t|     [95% Conf. Interval]
------------------------------------------------------+--------------------------------------------------------
                                  second_vaccination8 |  -1.488388   .4884515    -3.05   0.003    -2.454801   -.5219746
                                                      |
c.second_vaccination8#c.second_vaccination8 |   .0093579    .004245     2.20   0.029     .0009589    .0177568
                                                      |
                                                _cons |   64.14944   13.49103     4.75   0.000      37.4571    90.84177
---------------------------------------------------------------------------------------------------------------

81 . nlcom -_b[second_vaccination]/(2*_b[c.second_vaccination#c.second_vaccination])

       _nl_1:  -_b[second_vaccination]/(2*_b[c.second_vaccination#c.second_vaccination])

------------------------------------------------------------------------------
cases_~10000 |      Coef.   Std. Err.      z    P>|z|     [95% Conf. Interval]
-------------+----------------------------------------------------------------
       _nl_1 |   79.52607   11.48269     6.93   0.000     57.02041    102.0317
------------------------------------------------------------------------------

82 . outreg2 using Table4.xls, pval addstat(F-statistics, e(F))
   Table4.xls
   dir : seeout

83 . quietly display 79.52607^2  //6324.3958

84 . reg cases_per_10000 second_vaccination8 second_vaccination8_sq

      Source |       SS           df       MS      Number of obs   =       132
-------------+----------------------------------   F(2, 129)       =     13.83
       Model |  8480.12203         2  4240.06102   Prob > F        =    0.0000
    Residual |  39561.4831       129  306.678164   R-squared       =    0.1765
-------------+----------------------------------   Adj R-squared   =    0.1637
       Total |  48041.6052       131   366.72981   Root MSE        =    17.512

----------------------------------------------------------------------------------------
       cases_per_10000 |      Coef.   Std. Err.      t    P>|t|     [95% Conf. Interval]
-----------------------+----------------------------------------------------------------
   second_vaccination8 |  -1.488388   .4884514    -3.05   0.003    -2.454801   -.5219746
second_vaccination8_sq |   .0093579    .004245     2.20   0.029     .0009589    .0177568
                 _cons |   64.14944   13.49103     4.75   0.000      37.4571    90.84177
----------------------------------------------------------------------------------------
```

```
85 . margins, at(second_vaccination8== 79.52607 second_vaccination8_sq==6324.3958)

   Adjusted predictions                              Number of obs     =        132
   Model VCE    : OLS

   Expression   : Linear prediction, predict()
   at           : second_vac~8    =    79.52607
                  second_~8_sq    =    6324.396
```

	Margin	Delta-method Std. Err.	t	P>|t|	[95% Conf.	Interval]
_cons	4.966623	2.145781	2.31	0.022	.7211421	9.212103

```
86 . reg cases_per_10000 c.second_vaccination10##c.second_vaccination10
```

Source	SS	df	MS			
				Number of obs	=	132
				F(2, 129)	=	13.83
Model	8480.12209	2	4240.06104	Prob > F	=	0.0000
Residual	39561.4831	129	306.678163	R-squared	=	0.1765
				Adj R-squared	=	0.1637
Total	48041.6052	131	366.72981	Root MSE	=	17.512

cases_per_10000	Coef.	Std. Err.	t	P>|t|	[95% Conf.	Interval]
second_vaccination10	-1.525819	.50515	-3.02	0.003	-2.525271	-.5263676
c.second_vaccination10#c.second_vaccination10	.0093579	.004245	2.20	0.029	.0009589	.0177568
_cons	67.16365	14.44564	4.65	0.000	38.58259	95.7447

```
87 . nlcom -_b[second_vaccination10]/(2*_b[c.second_vaccination#c.second_vaccination10])

       _nl_1:  -_b[second_vaccination10]/(2*_b[c.second_vaccination#c.second_vaccination10])
```

cases_~10000	Coef.	Std. Err.	z	P>|z|	[95% Conf.	Interval]
_nl_1	81.52607	11.48269	7.10	0.000	59.02041	104.0317

```
88 . outreg2 using Table4.xls, pval addstat(F-statistics, e(F))
   Table4.xls
   dir : seeout

89 . quietly display 81.52607*81.52607 //6646.5001

90 . reg cases_per_10000 second_vaccination10 second_vaccination10_sq
```

Source	SS	df	MS			
				Number of obs	=	132
				F(2, 129)	=	13.83
Model	8480.12257	2	4240.06129	Prob > F	=	0.0000
Residual	39561.4826	129	306.67816	R-squared	=	0.1765
				Adj R-squared	=	0.1637
Total	48041.6052	131	366.72981	Root MSE	=	17.512

cases_per_10000	Coef.	Std. Err.	t	P>|t|	[95% Conf.	Interval]
second_vaccination10	**-1.525819**	**.50515**	**-3.02**	**0.003**	**-2.525271**	**-.5263678**
second_vaccination10_sq	**.0093579**	**.004245**	**2.20**	**0.029**	**.000959**	**.0177568**
_cons	**67.16365**	**14.44564**	**4.65**	**0.000**	**38.5826**	**95.7447**

91 . margins, at(second_vaccination10== 81.52607 second_vaccination10_sq==6646.5001)

```
Adjusted predictions                            Number of obs     =        132
Model VCE    : OLS

Expression   : Linear prediction, predict()
at           : second_va~10    =    81.52607
               second_~0_sq    =      6646.5
```

	Margin	Delta-method Std. Err.	t	P>|t|	[95% Conf.	Interval]
_cons	**4.96662**	**2.145781**	**2.31**	**0.022**	**.7211406**	**9.2121**

92 . //Table 5: Sensitivity Analysis of the First Vaccination

93 . //where 0, 2, 4, 6, 8, 10 percent of the city population develops natural Immunity.

94 . gen first_vaccination2=first_vaccination+2

95 . gen first_vaccination4=first_vaccination+4

96 . gen first_vaccination6=first_vaccination+6

97 . gen first_vaccination8=first_vaccination+8

98 . gen first_vaccination10=first_vaccination+10

99 . gen first_vaccination_sq=first_vaccination*first_vaccination

100 . gen first_vaccination2_sq=first_vaccination2*first_vaccination2

101 . gen first_vaccination4_sq=first_vaccination4*first_vaccination4

102 . gen first_vaccination6_sq=first_vaccination6*first_vaccination6

103 . gen first_vaccination8_sq=first_vaccination8*first_vaccination8

104 . gen first_vaccination10_sq=first_vaccination10*first_vaccination10

105 . reg cases_per_10000 c.first_vaccination##c.first_vaccination

Source	SS	df	MS
Model	**9964.96241**	**2**	**4982.48121**
Residual	**38076.6427**	**129**	**295.167773**
Total	**48041.6052**	**131**	**366.72981**

Number of obs	=	**132**
F(2, 129)	=	**16.88**
Prob > F	=	**0.0000**
R-squared	=	**0.2074**
Adj R-squared	=	**0.1951**
Root MSE	=	**17.18**

cases_per_10000	Coef.	Std. Err.	t	P>|t|	[95% Conf.	Interval]
first_vaccination	**-1.720879**	**.4404537**	**-3.91**	**0.000**	**-2.592328**	**-.8494308**
c.first_vaccination#c.first_vaccination	**.0120793**	**.0040865**	**2.96**	**0.004**	**.0039941**	**.0201645**
_cons	**66.7815**	**11.38998**	**5.86**	**0.000**	**44.24614**	**89.31686**

```
106 . outreg2 using Table5.xls,replace pval dec(4) addstat(F-statistics, e(F))
    Table5.xls
    dir : seeout

107 . nlcom -_b[first_vaccination]/(2*_b[c.first_vaccination#c.first_vaccination])

           _nl_1:  -_b[first_vaccination]/(2*_b[c.first_vaccination#c.first_vaccination])
```

cases_~10000	Coef.	Std. Err.	z	P>|z|	[95% Conf.	Interval]
_nl_1	**71.23255**	**7.252397**	**9.82**	**0.000**	**57.01811**	**85.44698**

```
108 . reg cases_per_10000 first_vaccination first_vaccination_sq
```

Source	SS	df	MS			
				Number of obs	=	**132**
				F(2, 129)	=	**16.88**
Model	**9964.96226**	**2**	**4982.48113**	Prob > F	=	**0.0000**
Residual	**38076.6429**	**129**	**295.167774**	R-squared	=	**0.2074**
				Adj R-squared	=	**0.1951**
Total	**48041.6052**	**131**	**366.72981**	Root MSE	=	**17.18**

cases_per_10000	Coef.	Std. Err.	t	P>|t|	[95% Conf.	Interval]
first_vaccination	**-1.720879**	**.4404537**	**-3.91**	**0.000**	**-2.592328**	**-.8494308**
first_vaccination_sq	**.0120793**	**.0040865**	**2.96**	**0.004**	**.0039941**	**.0201645**
_cons	**66.7815**	**11.38998**	**5.86**	**0.000**	**44.24614**	**89.31686**

```
109 . display  66.7815-1.720879*71.23256+.0120793*(71.23256^2)  //5.490189
    5.490189

110 . display 71.23255^2  //5074.0762
    5074.0762

111 . margins, at(first_vaccination== 71.23255 first_vaccination_sq==5074.0762)

    Adjusted predictions                              Number of obs     =        132
    Model VCE    : OLS

    Expression   : Linear prediction, predict()
    at           : first_vacc~n    =    71.23255
                   first_v~n_sq    =    5074.076
```

	Delta-method Margin	Std. Err.	t	P>|t|	[95% Conf.	Interval]
_cons	**5.490192**	**1.668317**	**3.29**	**0.001**	**2.189386**	**8.790999**

112 . reg cases_per_10000 c.first_vaccination2##c.first_vaccination2

Source	SS	df	MS
Model	9964.96226	2	4982.48113
Residual	38076.6429	129	295.167774
Total	48041.6052	131	366.72981

Number of obs	=	132
F(2, 129)	=	16.88
Prob > F	=	0.0000
R-squared	=	0.2074
Adj R-squared	=	0.1951
Root MSE	=	17.18

cases_per_10000	Coef.	Std. Err.	t	P>|t|	[95% Conf.	Interval]
first_vaccination2	-1.769196	.4564734	-3.88	0.000	-2.67234	-.8660525
c.first_vaccination2#c.first_vaccination2	.0120793	.0040865	2.96	0.004	.0039941	.0201645
_cons	70.27158	12.23702	5.74	0.000	46.06034	94.48281

113 . nlcom -_b[first_vaccination2]/(2*_b[c.first_vaccination2#c.first_vaccination2])

_nl_1: **-_b[first_vaccination2]/(2*_b[c.first_vaccination2#c.first_vaccination2])**

cases_~10000	Coef.	Std. Err.	z	P>|z|	[95% Conf.	Interval]
_nl_1	73.23255	7.252398	10.10	0.000	59.01811	87.44699

114 . outreg2 using Table5.xls, pval dec(4) addstat(F-statistics, e(F))
Table5.xls
dir : seeout

115 . quietly display 73.23255^2 //5363.0064

116 . reg cases_per_10000 first_vaccination2 first_vaccination2_sq

Source	SS	df	MS
Model	9964.96184	2	4982.48092
Residual	38076.6433	129	295.167778
Total	48041.6052	131	366.72981

Number of obs	=	132
F(2, 129)	=	16.88
Prob > F	=	0.0000
R-squared	=	0.2074
Adj R-squared	=	0.1951
Root MSE	=	17.18

cases_per_10000	Coef.	Std. Err.	t	P>|t|	[95% Conf.	Interval]
first_vaccination2	-1.769196	.4564734	-3.88	0.000	-2.67234	-.8660524
first_vaccination2_sq	.0120793	.0040865	2.96	0.004	.0039941	.0201645
_cons	70.27157	12.23702	5.74	0.000	46.06034	94.48281

117 . margins, at(first_vaccination2==73.23255 first_vaccination2_sq==5363.0064)

Adjusted predictions   Number of obs   =   132
Model VCE   : **OLS**

Expression   : **Linear prediction, predict()**
at   : first_vacc~2   =   **73.23255**
               first_v~2_sq   =   **5363.006**

	Margin	Delta-method Std. Err.	t	P>|t|	[95% Conf.	Interval]
_cons	5.490192	1.668317	3.29	0.001	2.189386	8.790998

118 . reg cases_per_10000 c.first_vaccination4##c.first_vaccination4

Source	SS	df	MS			
Model	9964.96228	2	4982.48114	Number of obs	=	132
Residual	38076.6429	129	295.167774	F(2, 129)	=	16.88
				Prob > F	=	0.0000
Total	48041.6052	131	366.72981	R-squared	=	0.2074
				Adj R-squared	=	0.1951
				Root MSE	=	17.18

cases_per_10000	Coef.	Std. Err.	t	P>|t|	[95% Conf.	Interval]
first_vaccination4	-1.817514	.4725155	-3.85	0.000	-2.752397	-.8826301
c.first_vaccination4#c.first_vaccination4	.0120793	.0040865	2.96	0.004	.0039941	.0201645
_cons	73.85829	13.11953	5.63	0.000	47.90098	99.81559

119 . nlcom -_b[first_vaccination4]/(2*_b[c.first_vaccination4#c.first_vaccination4])

_nl_1: **-_b[first_vaccination4]/(2*_b[c.first_vaccination4#c.first_vaccination4])**

cases_~10000	Coef.	Std. Err.	z	P>|z|	[95% Conf.	Interval]
_nl_1	75.23255	7.252398	10.37	0.000	61.01811	89.44699

120 . outreg2 using Table5.xls, pval dec(4) addstat(F-statistics, e(F))
Table5.xls
dir : seeout

121 . quietly display 75.23255^2 //5659.9366

122 . reg cases_per_10000 first_vaccination4 first_vaccination4_sq

Source	SS	df	MS			
Model	9964.96279	2	4982.48139	Number of obs	=	132
Residual	38076.6424	129	295.16777	F(2, 129)	=	16.88
				Prob > F	=	0.0000
Total	48041.6052	131	366.72981	R-squared	=	0.2074
				Adj R-squared	=	0.1951
				Root MSE	=	17.18

cases_per_10000	Coef.	Std. Err.	t	P>|t|	[95% Conf.	Interval]
first_vaccination4	-1.817514	.4725155	-3.85	0.000	-2.752397	-.8826303
first_vaccination4_sq	.0120793	.0040865	2.96	0.004	.0039941	.0201645
_cons	73.85829	13.11953	5.63	0.000	47.90098	99.81559

123 . margins, at(first_vaccination4==75.23255 first_vaccination4_sq==5659.9366)

```
Adjusted predictions                            Number of obs     =        132
Model VCE    : OLS

Expression   : Linear prediction, predict()
at           : first_vacc~4    =    75.23255
               first_v~4_sq    =    5659.937
```

	Margin	Delta-method Std. Err.	t	P>|t|	[95% Conf.	Interval]
_cons	5.490191	1.668317	3.29	0.001	2.189385	8.790998

124 . reg cases_per_10000 c.first_vaccination6##c.first_vaccination6

Source	SS	df	MS
Model	9964.96228	2	4982.48114
Residual	38076.6429	129	295.167774
Total	48041.6052	131	366.72981

```
Number of obs     =        132
F(2, 129)         =      16.88
Prob > F          =     0.0000
R-squared         =     0.2074
Adj R-squared     =     0.1951
Root MSE          =      17.18
```

cases_per_10000	Coef.	Std. Err.	t	P>|t|	[95% Conf.	Interval]
first_vaccination6	-1.865831	.4885777	-3.82	0.000	-2.832494	-.8991679
c.first_vaccination6#c.first_vaccination6	.0120793	.0040865	2.96	0.004	.0039941	.0201645
_cons	77.54163	14.03719	5.52	0.000	49.7687	105.3146

125 . nlcom -_b[first_vaccination6]/(2*_b[c.first_vaccination6#c.first_vaccination6])

```
       _nl_1:  -_b[first_vaccination6]/(2*_b[c.first_vaccination6#c.first_vaccination6])
```

cases_~10000	Coef.	Std. Err.	z	P>|z|	[95% Conf.	Interval]
_nl_1	77.23255	7.252398	10.65	0.000	63.01811	91.44699

126 . outreg2 using Table5.xls, pval dec(4) addstat(F-statistics, e(F))
Table5.xls
dir : seeout

127 . quietly display 77.23255^2 //5964.8668

128 . reg cases_per_10000 first_vaccination6 first_vaccination6_sq

Source	SS	df	MS
Model	9964.9619	2	4982.48095
Residual	38076.6433	129	295.167777
Total	48041.6052	131	366.72981

```
Number of obs     =        132
F(2, 129)         =      16.88
Prob > F          =     0.0000
R-squared         =     0.2074
Adj R-squared     =     0.1951
Root MSE          =      17.18
```

cases_per_10000	Coef.	Std. Err.	t	P>|t|	[95% Conf.	Interval]
first_vaccination6	-1.865831	.4885777	-3.82	0.000	-2.832494	-.8991678
first_vaccination6_sq	.0120793	.0040865	2.96	0.004	.0039941	.0201645
_cons	77.54163	14.03719	5.52	0.000	49.7687	105.3146

```
129 . margins, at(first_vaccination6==77.23255 first_vaccination6_sq==5964.8668)

Adjusted predictions                            Number of obs     =        132
Model VCE    : OLS

Expression   : Linear prediction, predict()
at           : first_vacc~6    =    77.23255
               first_v~6_sq    =    5964.867
```

	Margin	Delta-method Std. Err.	t	P>|t|	[95% Conf.	Interval]
_cons	5.490191	1.668317	3.29	0.001	2.189385	8.790998

```
130 . reg cases_per_10000 c.first_vaccination8##c.first_vaccination8
```

Source	SS	df	MS
Model	9964.96229	2	4982.48115
Residual	38076.6429	129	295.167774
Total	48041.6052	131	366.72981

Number of obs	=	132
F(2, 129)	=	16.88
Prob > F	=	0.0000
R-squared	=	0.2074
Adj R-squared	=	0.1951
Root MSE	=	17.18

cases_per_10000	Coef.	Std. Err.	t	P>|t|	[95% Conf.	Interval]
first_vaccination8	-1.914148	.5046581	-3.79	0.000	-2.912626	-.9156696
c.first_vaccination8#c.first_vaccination8	.0120793	.0040865	2.96	0.004	.0039941	.0201645
_cons	81.32161	14.98974	5.43	0.000	51.66405	110.9792

```
131 . nlcom -_b[first_vaccination8]/(2*_b[c.first_vaccination8#c.first_vaccination8])

       _nl_1:  -_b[first_vaccination8]/(2*_b[c.first_vaccination8#c.first_vaccination8])
```

cases_~10000	Coef.	Std. Err.	z	P>|z|	[95% Conf.	Interval]
_nl_1	79.23255	7.252398	10.93	0.000	65.01811	93.44699

```
132 . outreg2 using Table5.xls, pval dec(4) addstat(F-statistics, e(F))
    Table5.xls
    dir : seeout

133 . quietly display 79.23255^2  //6277.797

134 . reg cases_per_10000 first_vaccination8 first_vaccination8_sq

      Source |       SS           df       MS      Number of obs   =       132
-------------+----------------------------------   F(2, 129)       =     16.88
       Model |  9964.96233         2  4982.48116   Prob > F        =    0.0000
    Residual |  38076.6428       129  295.167774   R-squared       =    0.2074
-------------+----------------------------------   Adj R-squared   =    0.1951
       Total |  48041.6052       131   366.72981   Root MSE        =     17.18

------------------------------------------------------------------------------------
   cases_per_10000 |      Coef.   Std. Err.      t    P>|t|     [95% Conf. Interval]
-------------------+----------------------------------------------------------------
  first_vaccination8 |  -1.914148   .5046581    -3.79   0.000    -2.912626   -.9156696
first_vaccination8_sq |   .0120793   .0040865     2.96   0.004     .0039941    .0201645
             _cons |   81.32161   14.98974     5.43   0.000     51.66405    110.9792
------------------------------------------------------------------------------------

135 . margins, at(first_vaccination8==79.23255 first_vaccination8_sq==6277.797)

    Adjusted predictions                            Number of obs     =        132
    Model VCE    : OLS

    Expression   : Linear prediction, predict()
    at           : first_vacc~8    =    79.23255
                   first_v~8_sq    =    6277.797

------------------------------------------------------------------------------
             |            Delta-method
             |     Margin   Std. Err.      t    P>|t|     [95% Conf. Interval]
-------------+----------------------------------------------------------------
       _cons |   5.490191   1.668317     3.29   0.001     2.189385    8.790997
------------------------------------------------------------------------------

136 . reg cases_per_10000 c.first_vaccination10##c.first_vaccination10

      Source |       SS           df       MS      Number of obs   =       132
-------------+----------------------------------   F(2, 129)       =     16.88
       Model |  9964.96234         2  4982.48117   Prob > F        =    0.0000
    Residual |  38076.6428       129  295.167774   R-squared       =    0.2074
-------------+----------------------------------   Adj R-squared   =    0.1951
       Total |  48041.6052       131   366.72981   Root MSE        =     17.18

---------------------------------------------------------------------------------------------------------------
                                 cases_per_10000 |      Coef.   Std. Err.      t    P>|t|     [95% Conf. Interval]
-------------------------------------------------+-------------------------------------------------------------
                             first_vaccination10 |  -1.962465   .5207551    -3.77   0.000    -2.992792   -.9321386
                                                 |
c.first_vaccination10#c.first_vaccination10 |   .0120793   .0040865     2.96   0.004     .0039941    .0201645
                                                 |
                                           _cons |   85.19822   15.97692     5.33   0.000     53.58749     116.809
---------------------------------------------------------------------------------------------------------------
```

```
137 . nlcom -_b[first_vaccination10]/(2*_b[c.first_vaccination#c.first_vaccination10])

           _nl_1:  -_b[first_vaccination10]/(2*_b[c.first_vaccination#c.first_vaccination10])

------------------------------------------------------------------------------
cases_~10000 |      Coef.   Std. Err.      z    P>|z|     [95% Conf. Interval]
-------------+----------------------------------------------------------------
       _nl_1 |   81.23255   7.252398    11.20   0.000     67.01811    95.44699
------------------------------------------------------------------------------

138 . outreg2 using Table5.xls, pval dec(4) addstat(F-statistics, e(F))
    Table5.xls
    dir : seeout

139 . quietly display  81.23255^2  //6598.7272

140 . reg cases_per_10000 first_vaccination10 first_vaccination10_sq

      Source |       SS           df       MS      Number of obs   =       132
-------------+----------------------------------   F(2, 129)       =     16.88
       Model |  9964.96211         2  4982.48106   Prob > F        =    0.0000
    Residual |   38076.643       129  295.167775   R-squared       =    0.2074
-------------+----------------------------------   Adj R-squared   =    0.1951
       Total |  48041.6052       131   366.72981   Root MSE        =     17.18

-------------------------------------------------------------------------------------
    cases_per_10000 |      Coef.   Std. Err.      t    P>|t|     [95% Conf. Interval]
--------------------+----------------------------------------------------------------
  first_vaccination10 |  -1.962465   .5207551    -3.77   0.000    -2.992792   -.9321385
first_vaccination10_sq |   .0120793   .0040865     2.96   0.004     .0039941    .0201645
              _cons |   85.19822   15.97692     5.33   0.000     53.58749    116.8089
-------------------------------------------------------------------------------------

141 . margins, at(first_vaccination10== 81.23255 first_vaccination10_sq==6598.7272)

    Adjusted predictions                            Number of obs     =        132
    Model VCE    : OLS

    Expression   : Linear prediction, predict()
    at           : first_vac~10    =    81.23255
                   first_v~0_sq    =    6598.727

------------------------------------------------------------------------------
             |            Delta-method
             |     Margin   Std. Err.      t    P>|t|     [95% Conf. Interval]
-------------+----------------------------------------------------------------
       _cons |   5.490191   1.668317     3.29   0.001     2.189384    8.790997
------------------------------------------------------------------------------

142 .
143 .
    end of do-file

144 .
```
